# Nutraceuticals Induced Changes in the Broiler Gastrointestinal Tract Microbiota

**DOI:** 10.1128/mSystems.01124-20

**Published:** 2021-03-02

**Authors:** Emese Tolnai, Peter Fauszt, Gabor Fidler, Georgina Pesti-Asboth, Endre Szilagyi, Aniko Stagel, Jozsef Konya, Judit Szabo, Laszlo Stundl, Laszlo Babinszky, Judit Remenyik, Sandor Biro, Melinda Paholcsek

**Affiliations:** a Department of Human Genetics, Faculty of Medicine, University of Debrecen, Debrecen, Hungary; b Institute of Food Technology, Faculty of Agricultural and Food Sciences and Environmental Management, University of Debrecen, Debrecen, Hungary; c Department of Medical Microbiology, Faculty of Medicine, University of Debrecen, Debrecen, Hungary; d Department of Feed and Food Biotechnology, Institute of Animal Science, Biotechnology and Nature Conservation, Faculty of Agricultural and Food Sciences and Environmental Management, University of Debrecen, Debrecen, Hungary; University of Connecticut

**Keywords:** antibiotic-free meat production system, symbiotic-dysbiotic microbiota, bacterial 16S rRNA gene sequencing, carotenoids, anthocyanins, fermentable oligosaccharides, probiotics

## Abstract

Effects of nutraceuticals on the intestinal microbiota are receiving increased attention; however, there are few studies investigating their effects on broiler meat production. The aim of this study was to implement feeding strategies and carry out a comprehensive trial examining the interplay between natural biologically active compounds such as carotenoids, anthocyanins, fermentable oligosaccharides, and synbiotics and the gastrointestinal tract microbiota. Our feeding program was applied to an intensive production system with a flock of 1,080 Ross 308 broilers. Aging induced significant changes through the feeding experiment. Nutraceuticals were shown to modulate broiler intestinal diversity and differentially enriched *Lactobacillus*, *Enterococcus*, *Campylobacter*, and *Streptococcus* in the core microbiome during the different stages of broiler rearing. Additionally, they did not remarkably affect animal growth performance; nevertheless, a positive correlation was found between body weight and *Corynebacteriales* and *Pseudomonadales*. Furthermore, a diet high in carotenoid, fermentable oligosaccharide, and anthocyanin contents affected the number of beneficial genera such as *Faecalibacterium*, *Lactobacillus*, *Blautia*, and *Ruminococcus*. With this comprehensive trial, we revealed that nutraceuticals induced modulations in broiler gastrointestinal tract microbiota. We believe that plant-derived immunostimulants, recycled from plant food waste products, can supplement antibiotic-free broiler meat production.

**IMPORTANCE** In this trial, nutraceuticals were manufactured from waste products of food industry processing of Hungarian red sweet pepper and sour cherry and incorporated into the diet of poultry to investigate their effects on broilers’ growth and the broiler gastrointestinal tract microbiota. To avoid the generation of food waste products, we believe that this approach can be developed into a sustainable, green approach that can be implemented in commercial antibiotic-free poultry to provide safe and high-quality meat.

## INTRODUCTION

During the past 2 decades, the poultry industry has become one of the most efficient protein production systems, and it forms the basis of global protein production (1). Intensive breed selection was invented to develop chickens that convert feed into muscle mass more efficiently (2). Modern chicken breeds such as Ross 308 require less forage to achieve their desired increase (approximately 70 to 80×) in weight (35 g to ∼3 kg) throughout the production period (35 to 42 days) (3). This extreme growth rate can be associated with a range of pathological conditions (3–5), including hypertension, heart failure, insulin resistance, and increased susceptibility to infections (6–8).

The gastrointestinal tract (GIT) microbiota plays an important role in the overall health and function of the host (9–11). The GIT microbiota is the focus of major research efforts in meat production animals (12) since it has a positive impact on the immune system (12–14), GIT physiology (14, 15), nutrition (11, 16), and detoxification of certain compounds and productivity (16, 17). It also has an important role in the poultry industry, requiring animals capable of growing rapidly (18, 19).

There is growing evidence that alterations in poultry GIT microbiota composition have a pivotal role in the development of metabolic disorders (15, 20, 21). The diversity of the microbiota is one of the key determinants in resistance to invading pathogens (22). Higher microbial community diversity is related to a healthier host status, whereas a significant loss in complexity is associated with various diseases and susceptibility to pathogen colonization (16, 23–25). Shifts of the GIT microbiota toward beneficial bacteria could improve the health conditions of the host.

Through the past 80 years, antibiotics have been widely used to support the immunocompetence of birds against infectious diseases (26, 27). For animals that grow to a great degree, application of a subtherapeutic dose of antibiotics was generally shown to improve health and productivity (28). The routine and irresponsible use of such additives is associated with undesired consequences, such as depletion of the beneficial intestinal microbiota and emergence of antibiotic-resistant microbial pathogens (29, 30). The lateral exchange of genetic material across bacteria contributes to the spread of antimicrobial resistance and broadly disseminates harmful, antibiotic-resistant bacteria across the globe. This dramatic impact has been a serious threat to both human and veterinary medicine (31). Antibiotic resistance was identified by the World Health Organization (WHO) as one of the most significant global threats to public health, and their use as growth promoters was banned by the European Union (32, 33).

Health-promoting probiotic bacteria can ferment prebiotics that are undigestible and nonabsorbable for the host and convert them to lactic acid and short-chain fatty acids (SCFAs) (33–38). SCFA-producing bacteria may directly enhance the absorption of some nutrients and hence have a direct influence on metabolic functions. (39–41). It was already proven that the deterioration of community diversity and the associated alterations in SCFAs can be restored by alternative treatment strategies in both humans and animals (42), some of which may alleviate disease symptoms (36). These probiotic-based dietary supplements are increasingly considered to be effective in replacing antibiotics (43, 44). Furthermore, it is also suggested that a probiotic-enriched diet influences the intestinal absorption of broilers, thus improving production performance (45). Additionally, numerous studies emphasize the importance of prebiotic fibers, which can enhance the effects of live beneficial microorganisms (e.g., lactic acid bacteria; *Lactobacillus* and *Bifidobacterium*) (46).

Herbal medicines are receiving widespread attention, especially in developing countries, because of their antibacterial properties and improvement of performance and food safety (36, 37, 47–51). There is growing evidence that complex, bioactive compound-rich plant extracts increase digestive enzyme secretion and nutrient absorption and decrease the feed-to-gain ratio in meat-type chickens (15, 18, 48, 52–58).

More recently, nutraceuticals have become the focus of farm animal production. These nutraceuticals are rich in plant-derived immune stimulants such as phytochemicals, vitamins, and minerals (59). Several pre-, pro-, and synbiotic-based functional medicines have already been explored thoroughly and have demonstrated the ability to rebalance dysbiotic intestinal flora and preserve animal health (60). In this trial, we focused on natural, bioactive compounds (carotenoids, anthocyanins, functional oligosaccharides, and synbiotics) obtained from reprocessed plant-based food industrial waste materials and investigated their modulatory effect on the broiler gastrointestinal tract.

By enriching the diet of a flock of 1,080 Hungarian broilers with nutraceuticals, we investigated their effect on microbiota community diversity and alterations in the baseline symbiotic microbiota. We also managed to unravel compositional shifts in the GIT microbiota and investigated how these might relate to the growth performance of Ross 308 broilers.

## RESULTS

### General description of sequencing results.

The 16S rRNA gene-based (V3-V4 region) amplicon sequencing was carried out on the Illumina MiSeq platform, generating a total of ∼11 million reads by processing 96 broiler fecal samples with a mean count of 86,470 ± 24,361 reads per sample. Quality filtering with the DADA2 software resulted in an average denoised read count of 42,763 ± 13,425 per sample, and after a merging process, the read count dropped to an average of 41,085 ± 12,991 reads per sample. At the end, the average number of nonchimeric reads was 27,778 ± 7,622 per sample.

### Effects of nutraceuticals on body weight.

**FIG 1:**
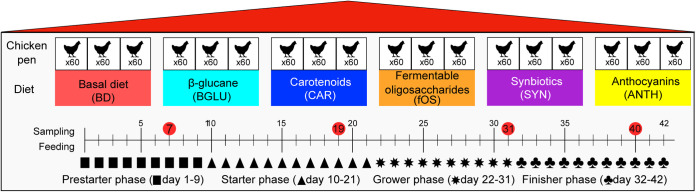
Overview of feeding and sampling strategies. Broiler chickens were fed a commercial maize-soybean-based basal diet (BD) that was formulated for prestarter (days 1 to 9), starter (days 10 to 21), grower (days 22 to 31), and finisher (days 32 to 42) production periods. BD negative control (basal diet with no dietary supplement) and the following dietary treatments were provided as mash feed: BGLU positive control (BD including β-glucan), CAR (BD including carotenoids), fOS (BD including fermentable oligosaccharides), SYN (BD including synbiotics), and ANTH (BD including anthocyanins).

The effects of dietary supplements on broiler growth body weight (BW) were monitored throughout the feeding trial (Fig. 1). At the beginning, the average BW values for birds were 38.4 ± 1.6 g, while by the end of this experiment broiler chicken reached 2,693 ± 64.8 g on average (see Table S1 in the supplemental material). No significant differences were noticed in body weight when comparing treatment groups (carotenoid [CAR], fermentable oligosaccharide [fOS], synbiotic [SYN], anthocyanin [ANTH]) to controls (basal diet [BD], β-glucan [BGLU]). However, by the end of the broiler productive life span, a moderate but not significant decrease in body weight was registered due to anthocyanin-based dietary supplementation in comparison to controls (ANTH BW, 2,590 ± 264 g, versus BD and BGLU BW, 2,742 ± 222 g).

10.1128/mSystems.01124-20.5 TABLE S1 Effects of bioactive compounds on body weight (g/bird) of Ross 308 broiler chickens at different time points. Download Table S1, PDF file, 0.09 MB. Copyright © 2021 Tolnai et al. 2021 Tolnai et al. https://creativecommons.org/licenses/by/4.0/ This content is distributed under the terms of the Creative Commons Attribution 4.0 International license.

### Significant associations were found between broiler body weight and the GIT microbiota.

**FIG 2:**
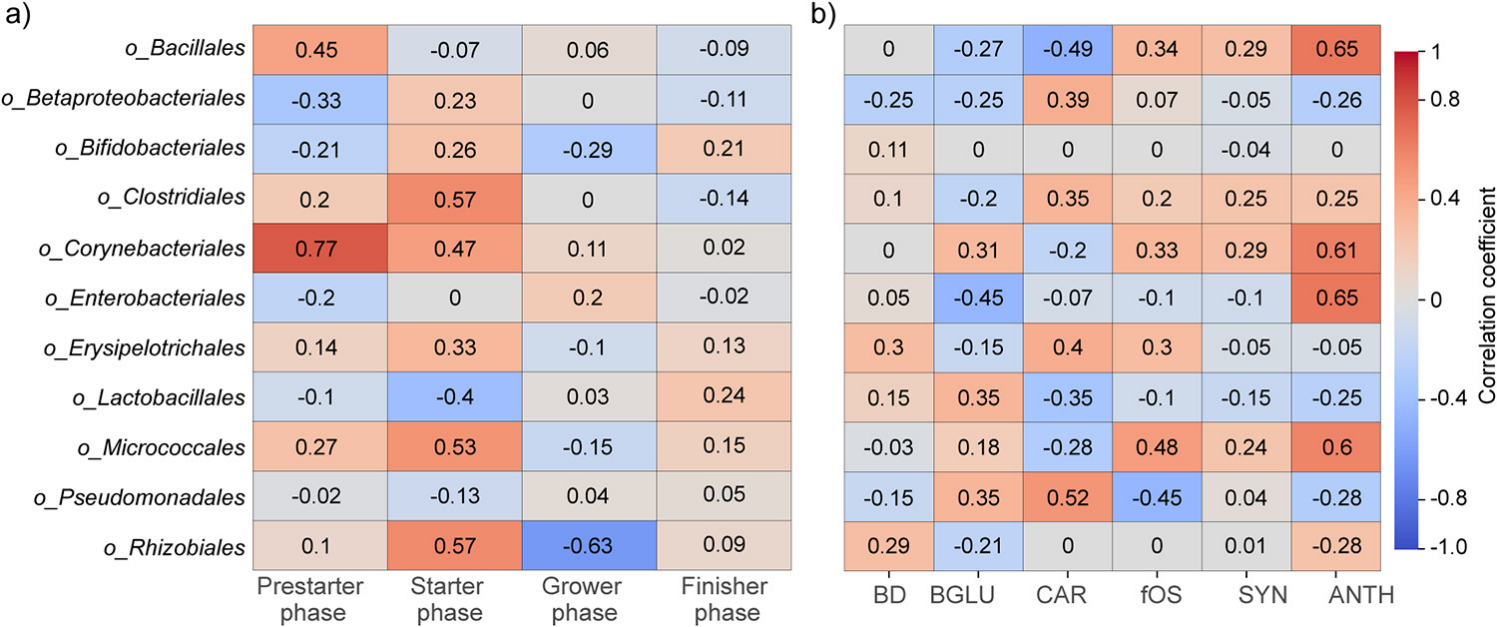
Spearman correlations were calculated to measure the extent of aging (prestarter, starter, grower, finisher) (a)- and diet (BD, BGLU, CAR, fOS, SYN, ANTH) (b)-related associations between BW and orders in the broiler GIT microbiota. The values of correlations varied from −1 to +1 and indicated the strength of positive (*R* ≥ 0; red) and negative (*R* < 0; blue) correlations. BD negative control (basal diet with no dietary supplement) and the following dietary treatments were provided as mash feed: BGLU positive control (BD including β-glucan), CAR (BD including carotenoids), fOS (BD including fermentable oligosaccharides), SYN (BD including synbiotics), and ANTH (BD including anthocyanins).

We managed to unravel alterations induced by age (prestarter, starter, grower, finisher) and treatment (BD, BGLU, CAR, fOS, SYN, ANTH) for 11 orders in the intestinal microbiota of Ross 308 broilers, finding remarkable correlations with body weight (Fig. 2). Alterations in the strengths and directions of correlations were obtained. In this study, out of the 11 orders, there were 6 (*Bacillales*, *Clostridiales*, *Corynebacteriales*, *Enterobacteriales*, *Micrococcales*, *Rhizobiales*) where moderate positive (age- and/or diet-specific) associations were detected with BW (*r* value > 0.4). We estimated that during the first two phases of the feeding experiment strong and/or moderate positive correlations were found between BW and the orders *Corynebacteriales*, *Bacillales*, *Clostridiales*, and *Micrococcales* (Fig. 2a). Interestingly, in the case of the order *Rhizobiales*, adverse, age-dependent correlations were found between starter and grower phases. In the case of the finisher phase, only weak or very weak correlations were found. When examining the effect of diet alone on the correlation values of these orders, we found strong positive (*r* value > 0.6) associations between the orders *Bacillales*, *Corynebacteriales*, *Enterobacteriales*, and *Micrococcales* and BW in ANTH-treated birds (Fig. 2b). Moderate negative (*r* value < −0.4) correlations were found between BW and *Enterobacteriales* in BGLU-treated birds. In the case of fOS-treated samples, moderate positive correlations were found with *Micrococcales*. Interestingly, *Pseudomonadales* exhibited moderate positive correlations with BW under CAR treatment and moderate negative correlations in fOS-treated samples. For the order *Bacillales*, moderate negative associations were shown in the CAR group.

### Age and treatment induced alterations in alpha and beta diversities.

**FIG 3:**
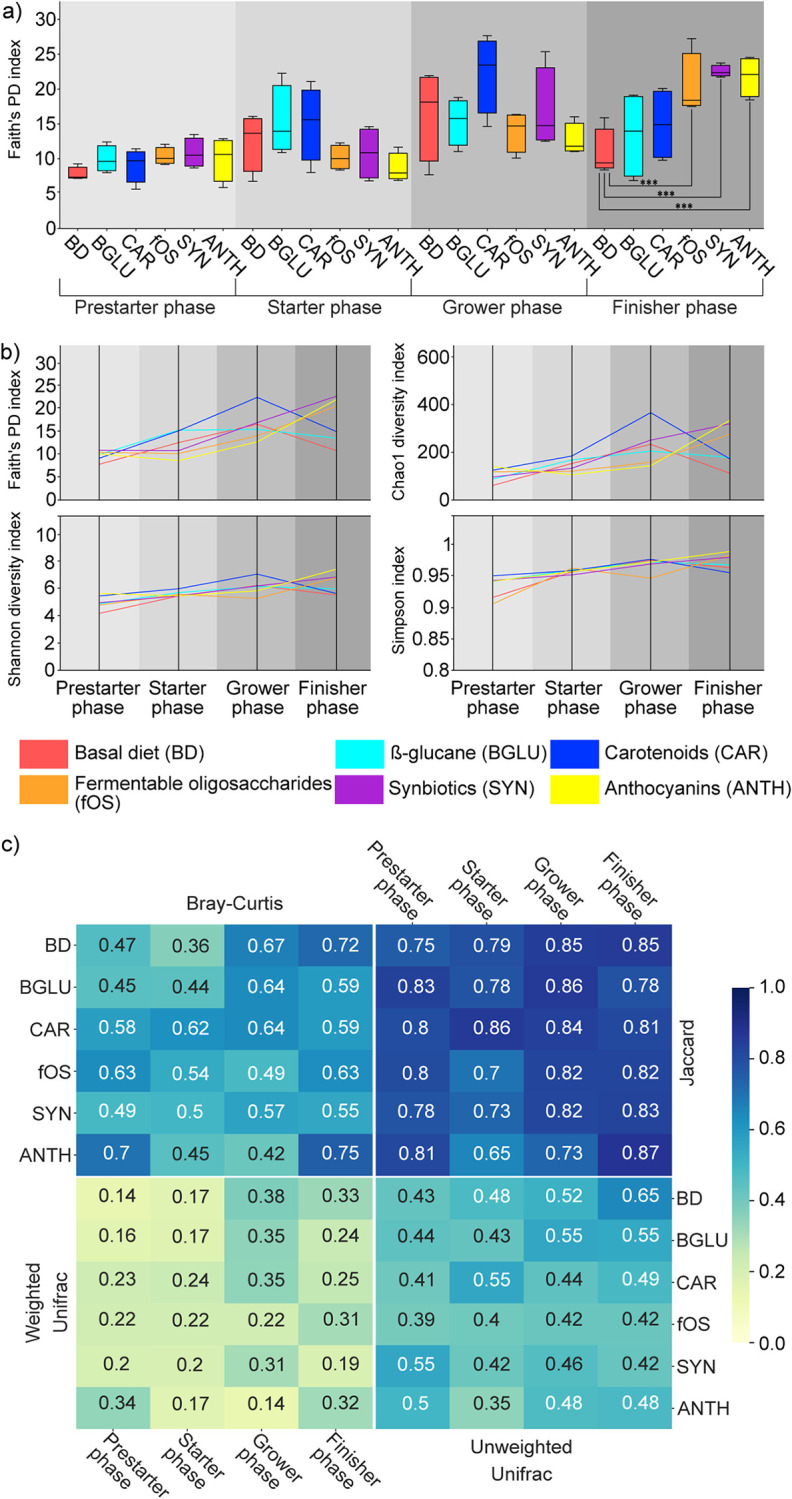
Community diversity distributions represent differences within and between our experimental groups. Statistical comparisons among multiple groups were performed with the nonparametric Kruskal-Wallis test, and intergroup differences were tested with Dunn’s test. (a) Boxplots represent comparisons of an alpha diversity metric (Faith’s PD diversity index) measured in different experimental groups. BD negative control (basal diet with no dietary supplement) and the following dietary treatments were provided as mash feed: BGLU positive control (BD including β-glucan), CAR (BD including carotenoids), fOS (BD including fermentable oligosaccharides), SYN (BD including synbiotics), and ANTH (BD including anthocyanins). Asterisks indicate statistical significance: ***, *P* ≤ 0.001. (b) Line graphs display the age-specific tendential changes in alpha diversity metrics observed in six experimental groups colored accordingly. The data shown are the mean values. (c) Sample distances were calculated on the basis of quantitative (Bray-Curtis, weighted UniFrac) and qualitative (Jaccard, unweighted UniFrac) dissimilarity-based statistics.

Both alpha and beta diversity indices were determined to track remarkable conversions in community diversities of control (BD, BGLU) and treatment (CAR, fOS, SYN, ANTH) groups (Fig. 3). Faith’s phylogenetic (Fig. 3a), Chao-1, Shannon, and Simpson (data not shown) diversity indices were applied to evaluate the species abundance, richness, and evenness of the broiler GIT microbiota. Faith’s phylogenetic diversity (PD) indicated a significant increase in chicken GIT community diversity by the end of the productive life span (finisher phase), in the cases of fermentable oligosaccharide- (fOS Faith’s PD: 20.3 ± 4.6), synbiotic- (SYN Faith’s PD: 22.5 ± 0.8), and anthocyanin-treated (ANTH Faith’s PD: 21.8 ± 2.9) birds in comparison to those receiving basal diet (BD Faith’s PD: 11.2 ± 4.0) (Fig. 3a). During the grower and finisher feeding phases, fOS, SYN, and ANTH treatment caused notable increases in Faith’s PD indices. Shannon and Simpson diversity indices did not show significant changes throughout the experiment due to nutraceuticals. In general, certain differences in pattern dynamics were observed in alpha diversity indices (Fig. 3b). Faith’s PD, Chao-1, Shannon, and Simpson indices improved steadily with animal growth, while a deterioration was observed in these parameters during the finisher phase of the experiment. Broadly, during the grower phase, the highest community diversity was associated with CAR-treated birds, while by the end of the finisher period the community diversity proved to be the lowest in the case of animals receiving basal diet. Four beta diversity heatmaps were generated by measuring Bray-Curtis, Jaccard, and weighted and unweighted UniFrac distances (Fig. 3c) between the different experimental groups in relation to age and diet. Distance-based dissimilarity matrices showed that flock development exerted a substantial influence on overall community variations; thus, a gradual increase in community diversity was accompanied by increased heterogeneity of the GIT microbiota.

### The baseline GIT microbiota reflects a dynamic equilibrium in livestock.

**FIG 4:**
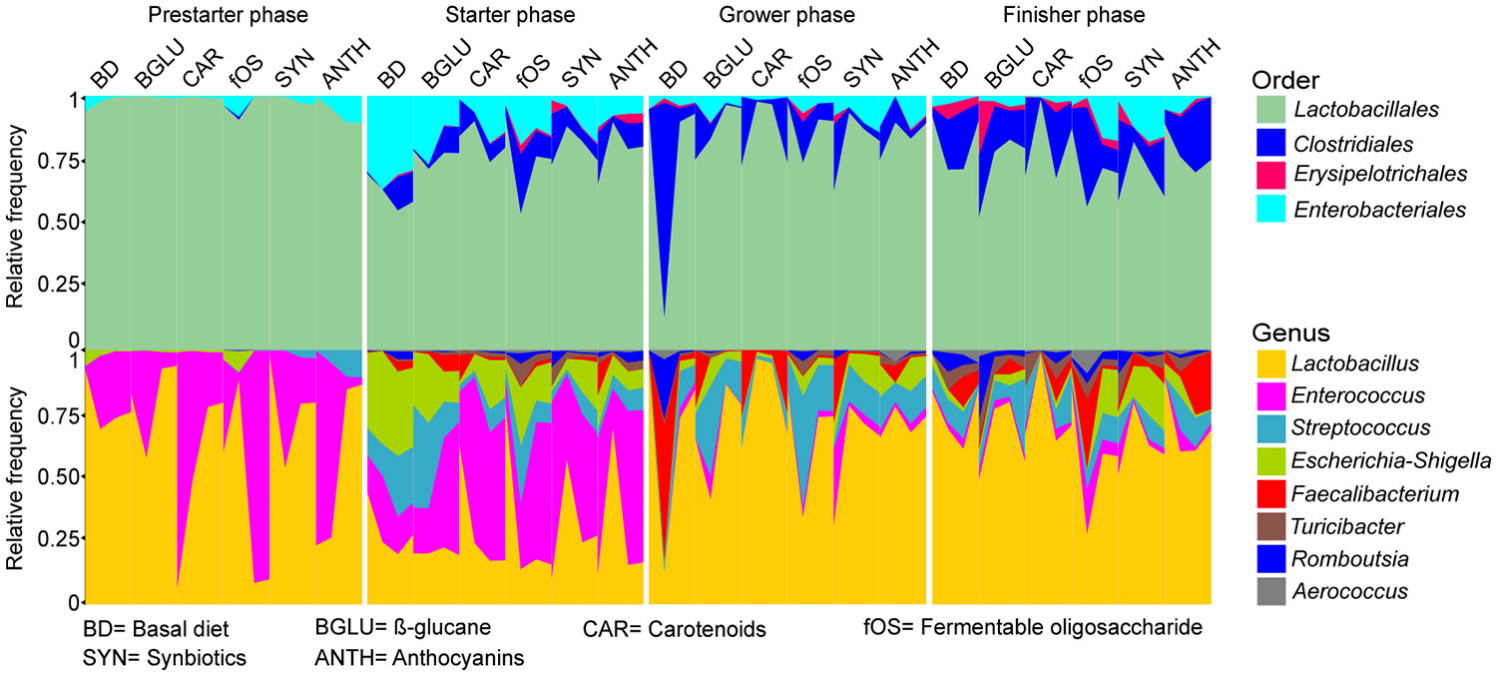
Variations in the healthy core 50% GIT microbiota of broilers over time. Area plots visualizing the core orders and genera according to age and diet. BD negative control (basal diet with no dietary supplement) and the following dietary treatments were provided as mash feed: BGLU positive control (BD including 0.5% β-glucan), CAR (BD including 0.5% carotenoids), fOS (BD including 0.5% fermentable oligosaccharides), SYN (BD including 0.5% synbiotics), and ANTH (BD including 0.5% anthocyanins).

Estimations of the healthy core microbiota were made for all experimental groups at the phylum, order, and genus taxonomic ranks, by considering taxa (order: 4; genus: 8) represented in at least 50% of the samples (Fig. 4). Characteristically, fermentable oligosaccharides, synbiotics, and anthocyanins exerted greatest community shifts in the core microbiota of starter chickens.

*Lactobacillales* was the most abundant order during the grower period (83.2% ± 17.7%) followed by *Clostridiales* (11.3% ± 17.7%), *Enterobacteriales* (4.9% ± 4.3%), and *Erysipelotrichales* (0.5% ± 0.9%). In the case of *Lactobacillales*, the highest relative abundances accounted for the prestarter feeding period (97.5% ± 3.3%). A relatively lower proportion of *Clostridiales* was shown in grower animals receiving immunostimulants in the form of β-glucan (BGLU: 7.4% ± 10.4%), fermentable oligosaccharides (fOS: 7.7% ± 6.0%), and anthocyanins (ANTH: 7.0% ± 5.2%) in comparison to those receiving the basal diet (BD: 23.3% ± 40.4%).

We found eight genera representing the core microbiota of at least 50% of samples: *Lactobacillus* (Σ55.7% ± 27.3%), *Enterococcus* (Σ19.0% ± 23.8%), *Streptococcus* (Σ7.7% ± 8.6%), *Escherichia-Shigella* (Σ6.9% ± 7.9%), *Faecalibacterium* (Σ3.5% ± 7.1%), *Turicibacter* (Σ1.1% ± 2.5%), *Romboutsia* (Σ1.7% ± 2.6%), and *Aerococcus* (Σ0.5% ± 1.1%). The genus *Lactobacillus* showed a clear dominance during the experiment except in starter samples (starter: 27.4% ± 19.1%) where the relative abundances shifted significantly in favor of *Enterococcus* (starter: 36.5% ± 18.3%). At the genus level, chicken development exerted the most explicit effect on the relative occurrence of *Enterococcus*. In young chickens, this genus seemed to be the second most abundant (prestarter-starter: 34.7% ± 24.9%), whereas in older chicks a drastic fall (grower-finisher: 3.2% ± 4.9%) was observed. By the end of the broiler rearing period, variations in the 50% core were diminished with the exception of two genera; fermentable oligosaccharides increased the relative proportions of *Enterococcus* (finisher fOS, 6.8% ± 6.4%, versus other treatment groups, 2.5% ± 2.2%), while nutraceutical treatment generally increased *Faecalibacterium* (finisher CAR: 2.9% ± 3.5%; fOS: 7.3% ± 11.9%; SYN: 3.6% ± 2%; ANTH: 12.5% ± 10.7%) in comparison to their control levels (finisher BD-BGLU: 3.1% ± 0.4%).

### The most pronounced community taxonomy shifts occurred due to age.

**FIG 5:**
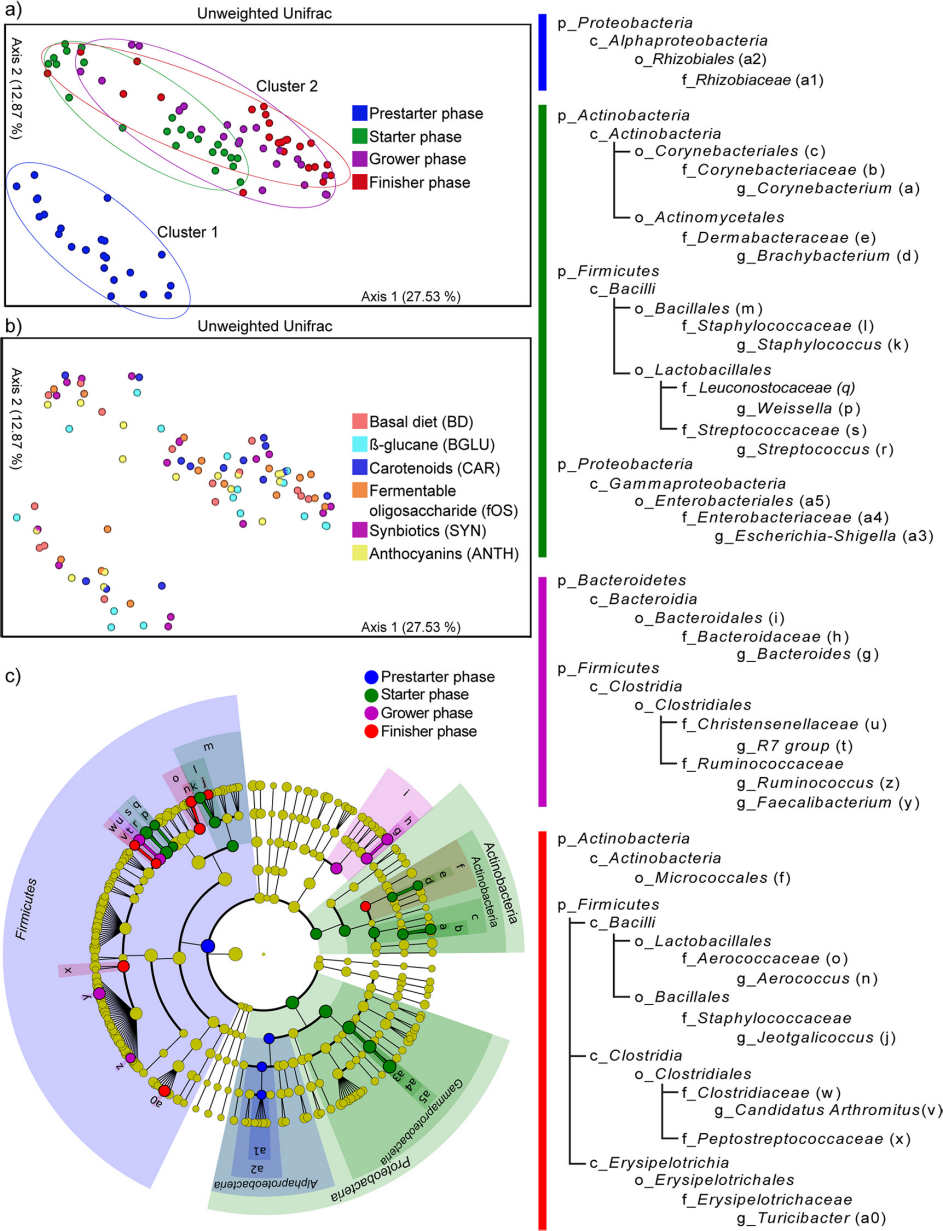
Beta diversity distributions summarizing the differences in community composition caused by aging (a) and diet (b). Beta diversity relationships are summarized in two-dimensional scatterplots. Each point represents a sample, and distances between dots are representative of differences in microbiota compositions. (c) Linear discriminant analysis effect size (LEfSe) identifies bacterial clades involved in significant taxonomic shifts. The cladogram depicts the phylogenetic distribution of microbial lineages in fecal samples obtained from broilers. (b) A list of 32 significantly enriched bacterial clades; including 6 orders, *Rhizobiales* (a2), *Enterobacteriales* (a5), *Corynebacteriales* (c), *Micrococcales* (f), *Bacillales* (m), and *Bacteroidales* (i); 12 families, *Rhizobiaceae* (a1), *Enterobacteriaceae* (a4), *Corynebacteriaceae* (b), *Dermabacteraceae* (e), *Bacteroidaceae* (h), *Staphylococcaceae* (l), *Aerococcaceae* (o), *Leuconostocaceae* (q), *Streptococcaceae* (s), *Christensenellaceae* (u), *Clostridiaceae* (w), and *Peptostreptococcaceae* (x); and 14 genera, *Corynebacterium* (a), *Turicibacter* (a0), *Escherichia-Shigella* (a3), *Brachybacterium* (d), *Bacteroides* (g), *Jeotgalicoccus* (j), *Staphylococcus* (k), *Aerococcus* (n), *Weissella* (p), *Streptococcus* (r), R7 group (t), “*Candidatus* Arthromitus” (v), *Faecalibacterium* (y), and *Ruminococcus* (z), organized with respect to diet and age. BD negative control (basal diet with no dietary supplement) and the following dietary treatments were provided as mash feed: BGLU positive control (BD including 0.5% β-glucan), CAR (BD including 0.5% carotenoids), fOS (BD including 0.5% fermentable oligosaccharides), SYN (BD including 0.5% probiotics), and ANTH (BD including 0.5% anthocyanins).

Beta diversity plots were made to investigate the age (Fig. 5a)- and diet (Fig. 5b)-induced alterations in community taxonomy. When measuring the age dependency of community taxonomy data with unweighted UniFrac metrics, principal-coordinate analysis (PCoA) resulted in two clusters (cluster 1 and cluster 2) representing different spatial ordinations between prestarter birds and older (starter, grower, and finisher) broilers (Fig. 5a). Furthermore, starter, grower, and finisher birds continued to cluster less separately (Fig. 5a). When marking the samples according to diet, no distinct patterns became apparent between the treatment groups (Fig. 5b). On the basis of the PCoA plots, we concluded that age exerted more pronounced community shifts than diet. Not surprisingly, the prestarter microbiota showed less variation among samples. Additionally, the prestarter microbiota clustered distinctly in comparison to the microbiota at later time points of the experiment.

To decipher key taxa representing significant shifts during different stages of broiler production, the differentially abundant linear discriminant analysis effect size (LEfSe) method was used to perform class comparisons among feeding phases in chickens without any treatment (BD) (Fig. 5c). We found 32 bacterial clades that were significantly enriched with respect to age. These clades included 6 orders, *Rhizobiales* (a2), *Enterobacteriales* (a5), *Corynebacteriales* (c), *Micrococcales* (f), *Bacillales* (m), and *Bacteroidales* (i); 12 families, *Rhizobiaceae* (a1), *Enterobacteriaceae* (a4), *Corynebacteriaceae* (b), *Dermabacteraceae* (e), *Bacteroidaceae* (h), *Staphylococcaceae* (l), *Aerococcaceae* (o), *Leuconostocaceae* (q), *Streptococcaceae* (s), *Christensenellaceae* (u), *Clostridiaceae* (w), and *Peptostreptococcaceae* (x); and 14 genera, *Corynebacterium*(a), *Turicibacter*(a0), *Escherichia-Shigella*(a3), *Brachybacterium*(d), *Bacteroides* (g), *Jeotgalicoccus* (j), *Staphylococcus* (k), *Aerococcus* (n), *Weissella* (p), *Streptococcus* (r), R7 group (t), “*Candidatu*s Arthromitus” (v), *Faecalibacterium* (y), and *Ruminococcus* (z). During prestarter phase, great increases were seen in the order *Rhizobiales*, and during starter phase, the orders *Corynebacteriales*, *Actinomycetales*, and *Enterobacteriales* increased remarkably. Notable gains in the orders *Bacillales* and *Lactobacillales* were seen during both starter and finisher stages. The order *Clostridiales* was enriched in grower and finisher birds. More accessions were identified in the order *Bacteroidales* during the grower phase. Finally, compelling rises were seen during the finisher phase in *Erysipelotrichales*.

### Immunostimulant-driven alterations in family taxonomy.

**FIG 6:**
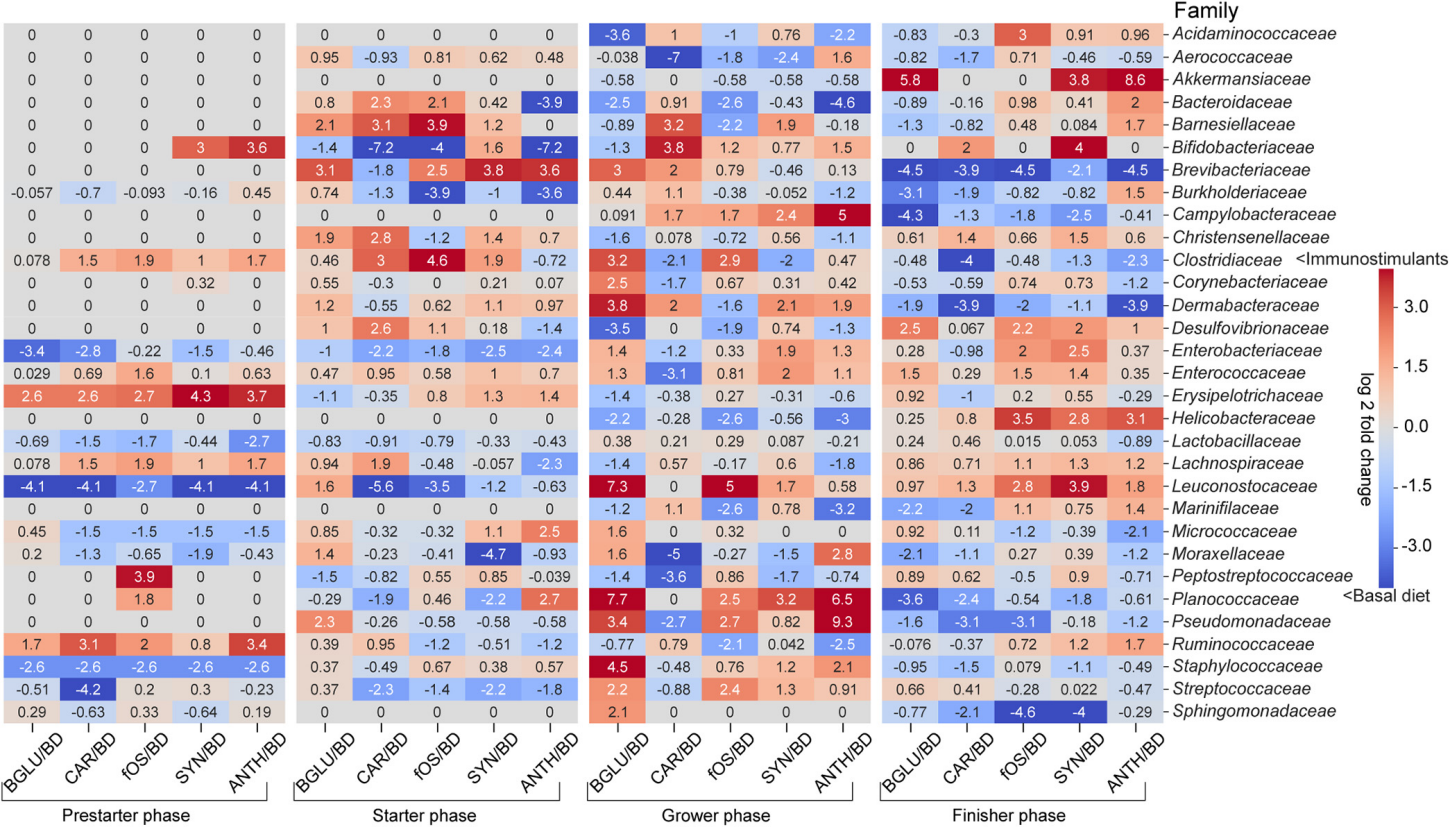
Annotated heatmap showing the extents of the estimated differences with the normalized log_2_ fold change of the specified family abundances (relative % frequencies > 0.1). The red scale represents the dominance of the family due to dietary supplementation, log_2_ (supplemented/nonsupplemented diet) > 0, whereas the blue scale represents values of increases in favor of negative controls, log_2_ (supplemented/nonsupplemented diet) < 0. BD negative control (basal diet with no dietary supplement) and the following dietary treatments were provided as mash feed: BGLU positive control (BD including 0.5% β-glucan), CAR (BD including 0.5% carotenoids), fOS (BD including 0.5% fermentable oligosaccharides), SYN (BD including 0.5% synbiotics), and ANTH (BD including 0.5% anthocyanins).

By considering the 31 most abundant families (relative % frequencies > 0.1), we managed to explore remarkable alterations in taxonomic data during the four phases of the feeding period when comparing BGLU- and nutraceutical-treated birds (CAR, fOS, SYN, and ANTH) to nontreated controls (BD). A composite heatmap was created to show distortions in the relative abundance data normalized to that of BD animals (Fig. 6). During the prestarter phase, we observed remarkable increases in *Bifidobacteriaceae* due to synbiotics and anthocyanins, while fOS supplementation resulted in higher levels of *Peptostreptococcaceae*. Nutraceuticals increased *Clostridiaceae* and *Lachnospiraceae*. Additionally, greater abundances were observed in *Erysipelotrichaceae* and *Ruminococcaceae* in anthocyanin-challenged animals. Immunostimulants decreased the levels of *Enterobacteriaceae*, *Leuconostocaceae*, and *Staphylococcaceae* in comparison to their levels in the negative control (BD). In fOS-treated starter birds, remarkable increases were shown in *Bacteroidaceae*, *Barnesiellaceae*, *Brevibacteriaceae*, and *Clostridiaceae* accompanied by decreases in *Bifidobacteriaceae* and *Burkholderiaceae*. During the grower phase, carotenoids increased *Barnesiellaceae* and *Bifidobacteriaceae* and decreased *Aerococcaceae*, *Clostridiaceae*, *Enterococcaceae*, *Moraxellaceae*, and *Peptostreptococcaceae*. In grower animals, solid increases in *Campylobacteraceae*, *Planococcaceae*, and *Pseudomonadaceae* and decreases in *Bacteroidaceae*, *Helicobacteraceae*, and *Marinifilaceae* were registered due to anthocyanins. In the finisher phase, impressive decreases were encountered in *Brevibacteriaceae* in all of the treatment groups. Enrichments in *Helicobacteraceae* occurred through fOS, SYN, and ANTH treatments. Additionally, an increase was detected in *Akkermansiaceae* due to BGLU, SYN, and ANTH.

### Alterations in the occurrence of SCFA-producing bacteria.

**FIG 7:**
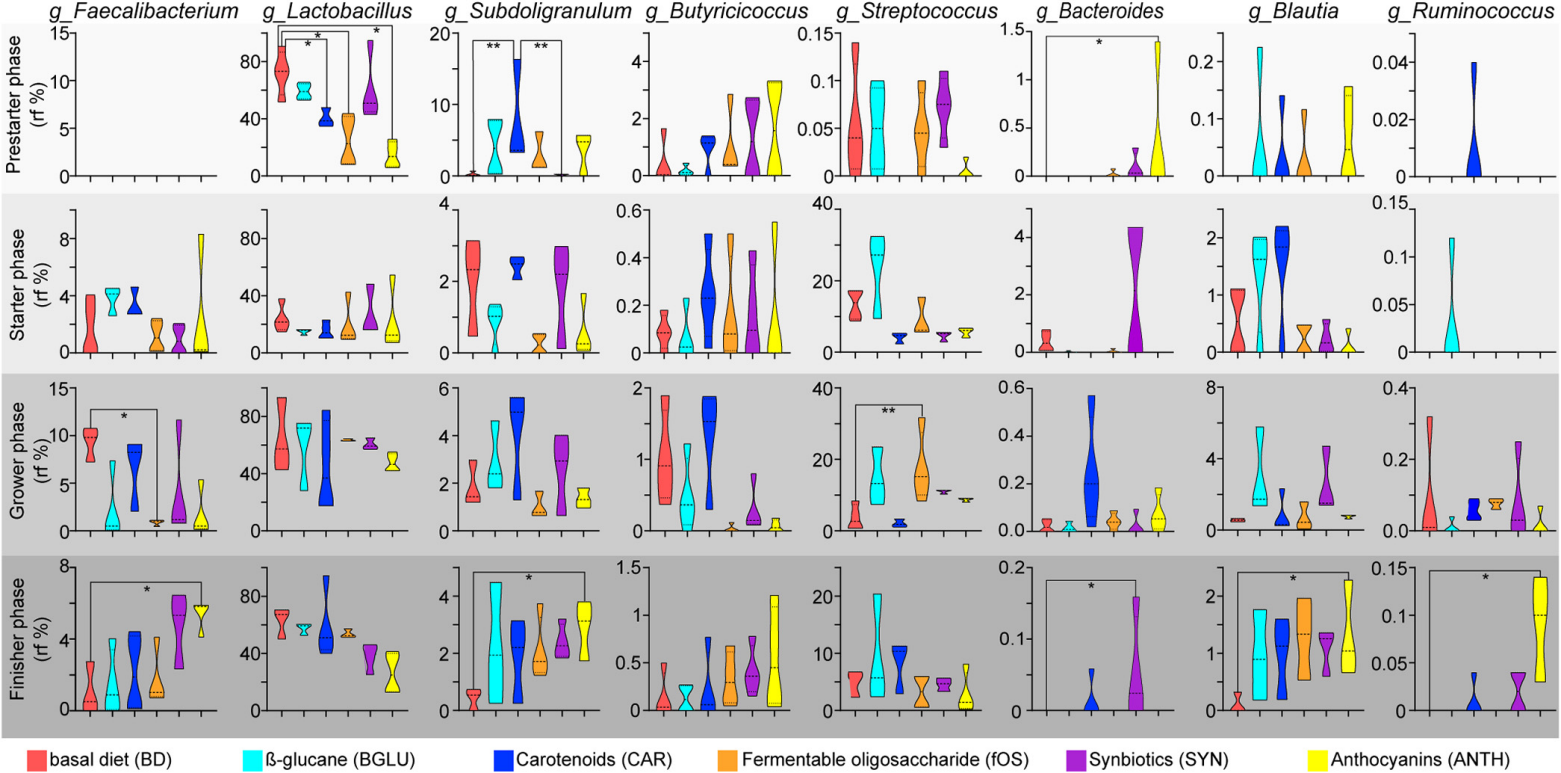
Shifts in the relative abundances of short-chain fatty acid-producing genera: *Faecalibacterium*, *Lactobacillus*, *Subdoligranulum*, *Butyricicoccus*, *Streptococcus*, *Bacteroides*, *Blautia*, and *Ruminococcus*. Age-related distributions of dedicated short-chain fatty acid-producing genera through four phases of broiler rearing: prestarter phase, starter phase, grower phase, and finisher phase. Violin plots show the influence of diet on the distribution of the short-chain fatty acid-producing genera. Asterisks indicate statistical significance: *, *P* ≤ 0.05; **, *P* ≤ 0.01. BD negative control (basal diet with no dietary supplement) and the following dietary treatments were provided as mash feed: BGLU positive control (BD including β-glucan), CAR (BD including carotenoids), fOS (BD including fermentable oligosaccharides), SYN (BD including synbiotics), and ANTH (BD including anthocyanins).

Among a range of metabolites produced by the beneficious gastrointestinal tract microbiota, short-chain fatty acids (SCFAs) have received increased attention because of their important role in disease prevention and recovery (61). In this trial, appreciable alterations were found in the proportions of some genera associated with SCFA production (Fig. 7).

The genus *Faecalibacterium* significantly decreased due to fOS in grower (fOS, 0.9 % ± 0.3%, versus BD, 9.3% ± 1.8%; *P* < 0.05) and increased due to ANTH in finisher (ANTH, 5.2% ± 1.4%, versus BD, 1.6% ± 1.6%; *P* < 0.05) chickens. The alterations in the relative frequencies of *Lactobacillus* (Σ38.8% ± 24.1%) were age rather than diet related; however, during prestarter phase, the genus *Lactobacillus* showed significantly (*P* < 0.05) higher levels in birds fed a basal diet (prestarter BD: 72.2% ± 15.9%) than in carotenoid (CAR: 31.1% ± 19.4%)-, fermentable oligosaccharide (fOS: 24.3% ± 18.1%)-, and anthocyanin (ANTH: 14.7% ± 9.0%)-treated animals. Fermentable oligosaccharide, synbiotic, and anthocyanin treatments had relative increases in several genera by grower phase. The elevating frequencies of *Lactobacillus* during the grower phase of broiler production might be associated with the antipathogenic characteristics of the members of this genus. The butyrate-producer genus *Subdoligranulum* increased significantly (*P* < 0.001) due to carotenoids in comparison to basal diet and synbiotics fed in prestarter animals (CAR, 5.8% ± 7.1%, versus BD, 0.2% ± 0.3%; SYN, 0.1% ± 0.1%). Further significant increase was detected in the relative frequency of *Subdoligranulum* due to anthocyanin treatment relative to birds fed basal diet in finisher birds (ANTH: 2.8% ± 1.6%; BD: 0.8% ± 0.9%; *P* < 0.05). Notably, the genera *Streptococcus*, *Blautia*, and *Ruminococcus* were barely (<0.25%) prevalent in prestarter birds. During the finisher stage of broiler meat production, anthocyanin treatment significantly increased the abundance of *Blautia* (finisher ANTH, 1.3% ± 0.4%, versus BD, 0.1% ± 0.2%) and *Ruminococcus* (finisher ANTH, 0.1% ± 0.05%, versus BD, 0% ± 0%) relative to those of the negative control. The synbiotics exerted a beneficial effect on the *Bacteroides* population. Remarkable increases in the relative proportions of this genus were found in starter (SYN, 2.2% ± 2.3%, versus other, 0.1% ± 0.3%) and finisher (SYN, 0.09% ± 0.08%, versus BD, 0%; *P* < 0.05) animals.

### Shifts in taxa involved in lipid metabolism.

**FIG 8:**
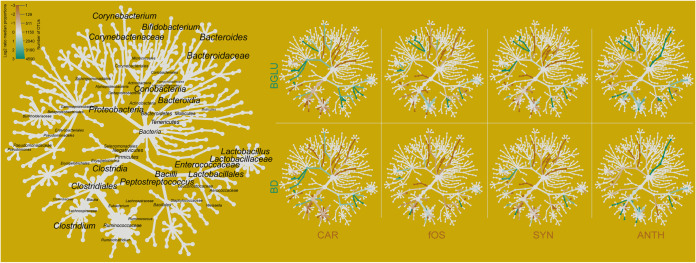
Differentially abundant taxonomic heat trees revealed the effects of nutraceuticals on taxa involved in lipid metabolism. The Metacoder differential heat tree illustrates the variation in microbiome phylotypes between experimental groups. The annotated tree on the left functions as a map for the unlabeled trees. Colored taxa represent the extents of log_2_ differences in taxon abundances: green represents higher abundance in BD or BGLU, while brown means higher abundance in nutraceutical-treated groups. Nodes in the heat tree correspond to phylotypes, as indicated by node labels, while edges link phylotypes in accordance with the taxonomic hierarchy. Node size corresponds to the number of operational taxonomic units (OTUs) observed within a given phylotype. BD negative control (basal diet with no dietary supplement) and the following dietary treatments were provided as mash feed: BGLU positive control (BD including 0.5% β-glucan), CAR (BD including 0.5% carotenoids), fOS (BD including 0.5% fermentable oligosaccharides), SYN (BD including 0.5% synbiotics), and ANTH (BD including 0.5% anthocyanins).

The intricate interconnections of the genera *Lactobacillus* (62, 63), *Enterococcus* (64), *Bifidobacterium* (65), *Clostridium* (66), *Bacteroides* (67), and *Peptostreptococcus* (68) regulate primary bile salt synthesis and secondary bile salt metabolism of the host (69). In order to investigate nutraceutical-induced community shifts connected to primary and secondary bile salt metabolism, taxonomic heat trees have been made in order to reveal effects of nutraceuticals on taxa involved in lipid metabolism (Fig. 8). Noticeably, ANTH decreased while CAR, fOS, and SYN increased the class *Bacteroidia* relative to BGLU and BD. ANTH decreased the relative abundance of the family *Lactobacillaceae* to those in both of the control groups (BD, BGLU). A slight increase was observed in *Enterococcaceae* frequencies due to nutraceuticals. Appreciable losses were detected in *Clostridium* due to fOS, SYN, and ANTH in comparison to BD.

### Diet-induced compositional differences can affect microorganisms involved in carbohydrate metabolism.

**FIG 9:**
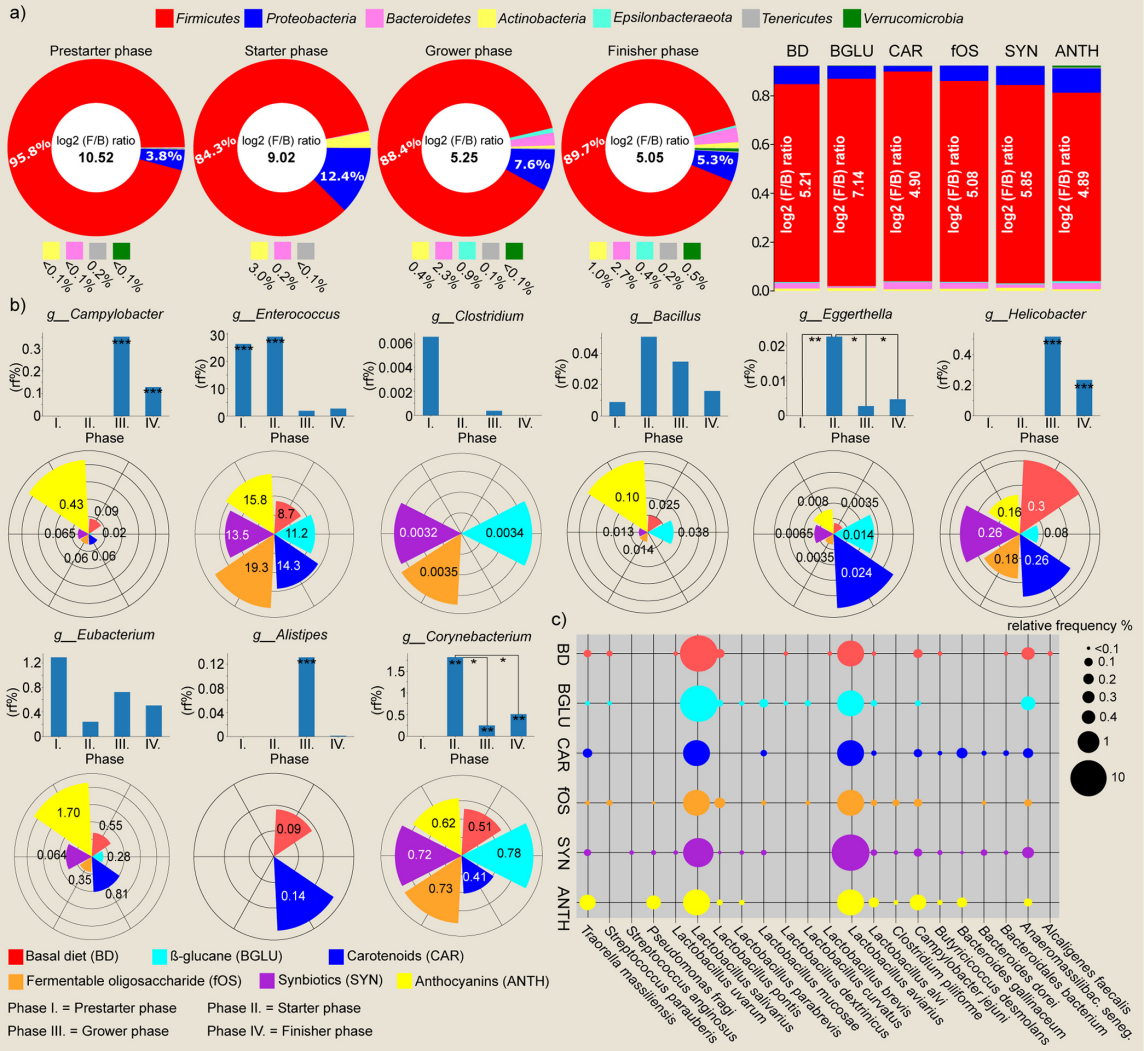
Shifts in taxa involved in carbohydrate metabolism. (a) Donut plots represent the diet-induced distortions in the main phyla. *Firmicutes*-to-*Bacteroides* ratios (log_2_ ratio of F/B relative % frequencies) are also indicated. (b) Bar charts represent rearing while polar plots (values in the pie portions indicate relative frequencies) show diet-related trends in relevant genera: *Campylobacter*, *Enterococcus*, *Clostridium*, *Bacillus*, *Eggerthella*, *Helicobacter*, *Eubacterium*, *Alistipes*, and *Corynebacterium* are involved in carbohydrate metabolism and pathogenesis. (c) Bubble chart showing 22 dedicated species, where bubble sizes correspond to relative abundance values. BD negative control (basal diet with no dietary supplement) and the following dietary treatments were provided as mash feed: BGLU positive control (BD including 0.5% β-glucan), CAR (BD including 0.5% carotenoids), fOS (BD including 0.5% fermentable oligosaccharides), SYN (BD including 0.5% synbiotics), and ANTH (BD including 0.5% of anthocyanins).

Both *Bacteroides* and *Firmicutes* are associated with SCFA synthesis (1). The end products of dietary fiber fermentation have been shown to exert multiple beneficial effects on mammalian energy metabolism by enhancing the absorption of some nutrients (39–41). According to previous publications, elevated *Firmicutes* levels can be associated with increased nutrient absorption, whereas *Bacteroidetes* enrichment usually correlates with enhanced hydrolysis of glycogen, starch, and polysaccharides promoting feed utilization and digestion of the host (1, 70, 71). The *Firmicutes-*to-*Bacteroidetes* (F/B) ratio is important for the optimal nutritional requirements of the host (56). Under our experimental settings, a total of 7 phyla were identified. Among these, *Firmicutes* (Σ89.5% ± 7.8%), *Proteobacteria* (Σ7.3% ± 7.0%), and *Bacteroidetes* (Σ1.3% ± 2.7%) were the most predominant, followed by *Actinobacteria*, *Proteobacteria*, *Tenericutes*, and *Verrucomicrobia*. F/B ratio was biased more by age than diet (Fig. 9a). Differences in the *Firmicutes*-to-*Bacteroides* ratios may reflect alterations in (poly)saccharide utilization of flocks. Characteristically, log_2_ F/B ratios represent a remarkable decrease in the course of the broiler production (prestarter phase, 10.52; starter phase, 9.02; grower phase, 5.25; finisher phase, 5.05). The values were highest during the prestarter phase and then decreased significantly by the end of the feeding trial (*P* < 0.05). The highest log_2_ F/B ratio was detected in BGLU birds, 7.14 (*Firmicutes*, 92.0%, versus *Bacteroides*, 0.7%), while it proved to be the lowest in anthocyanin-treated samples, 4.89 (*Firmicutes*, 83.6%, versus *Bacteroides*, 2.8%). Anthocyanins increased while carotenoids decreased the proportion of *Proteobacteria*. *Epsilonbacteraeota*, *Tenericutes*, and *Verrucomicrobia* were also detectable but with very low abundances (≤1%).

We also considered genera involved in carbohydrate metabolism that may include potential avian-pathogenic organisms (such as *Enterococcus* [72], *Clostridium* [24, 25], and *Helicobacter* [73]). The probiotic genera *Bacillus* and *Eubacterium* showed the highest occurrence for the treatment with ANTHs (Fig. 9b). Regarding its age-related distribution, the genus *Bacillus* was least abundant during the prestarter phase and reached its highest abundances during the starter phase (prestarter, 0.008% ± 0.02%; starter, 0.05% ± 0.14%) of the experiment, while the genus *Eubacterium* (prestarter, 1.3% ± 3.3%, versus others, 0.5% ± 0.7%) was the most abundant genus during the prestarter phase of the experiment. The genus *Corynebacterium*, which can include strains causing serious outbreaks of avian infections, was not detected during the prestarter phase but peaked at the starter phase (starter, 1.8% ± 0.6%, versus grower, 0.2% ± 0.5%; finisher, 0.5% ± 0.4%; *P* < 0.05). *Alistipes*, whose members are important in the fermentation of dietary fiber, was scarce in abundance during this experiment and detected during only the grower phase (grower: 0.1% ± 0.3%) and in birds receiving basal diet (BD: 0.09% ± 0.2%) and carotenoid (CAR: 0.1% ± 0.2%) supplementation. Our data indicated that in comparison to the basal diet, nutraceuticals had decreased relative abundance of *Helicobacter* (nutraceuticals, 0.2% ± 0.3%, versus BD, 0.3% ± 0.8%); anthocyanins increased the abundance of *Campylobacter* (ANTH, 0.4% ± 1.6%, versus other, 0.05% ± 0.1%), *Bacillus* (ANTH, 0.1 ± 0.1%, versus other, 0.01% ± 0.05%), and *Eubacterium* (ANTH, 1.7% ± 0.4%, versus other, 0.5% ± 0.6%); carotenoids increased *Eggerthella* (CAR, 0.02% ± 0.07%, versus other, 0.004% ± 0.01%); and the genus *Clostridium* was not detected in CAR- and ANTH-treated birds. We noticed a significant increase in *Campylobacter* and *Helicobacter* during the grower (*P* < 0.001) and finisher (*P* < 0.001) phases of the experiment. *Clostridium* was mainly detected during the prestarter phase. In the case of *Enterococcus*, a significant decrease was observed during the last two phases (grower-finisher, 2.3% ± 2.1%, versus prestarter-starter, 27.6% ± 21.4% *P* < 0.001) of the experiment. In the case of *Eggerthella*, significant increases (*P* < 0.01) were detected during the starter phase.

Attention was also paid to the estimated relative proportions of relevant species involved in lipid and carbohydrate metabolism, such as those involved in avian infections (Fig. 9c). Noticeably, β-glucan-treated samples showed the highest species diversity for lactic acid bacteria, covering eight *Lactobacillus* strains. Levels of the beneficial Lactobacillus aviarius and Lactobacillus salivarius, which is one of the main suppliers of the enzyme bile salt hydrolase (BSH) (74) and is also known to provide protection against colonization by *Salmonella* and other pathogens, were observed in all experimental groups. *L. salivarius* showed enrichment in the control animals (BD-BGLU, 15.2% ± 17.8%, versus nutraceutical groups, 7.5% ± 9.2%), whereas *L. aviarius* showed remarkable increases due to synbiotics (SYN: 14.7% ± 15.6%) and anthocyanins (ANTH: 6.8% ± 9.2%). Lactobacillus alvi, which is frequently obtained from chicken fecal and intestine (75), was also represented uniformly and showed increases in anthocyanin-treated samples (ANTH, 0.11% ± 0.2%, versus other groups, 0.01% ± 0.04%).

Noticeably, a rise in the bacterial diarrheal gastroenteritis-causing Campylobacter jejuni (ANTH, 0.5% ± 1.6%, versus others, 0.05% ± 0.1%) was shown in anthocyanin-fed animals without changes in chicken welfare. We detected the anaerobic Anaeromassilibacillus senegalensis having a short exposure time under aerobic conditions (76) in all of our experimental groups with a similar frequency (Σ0.1% ± 0.4%), which can reflect adequate sample handling and processing. Bacteroides gallinaceum, which was previously isolated from the ceca of a healthy broiler, seems to play an important role in the digestive system (77). However, it was traceable only in carotenoid (CAR: 0.1% ± 0.3%)- and anthocyanin (ANTH: 0.1% ± 0.2%)-treated samples. Butyrate-producing Butyricicoccus desmolans was traceable only in very low proportions in all sample sets. The lowest level of the newly described anaerobic, non-spore-forming, fatty acid-producing Traorella massiliensis (76) was observed in higher abundance among birds treated with anthocyanin (ANTH, 0.4% ± 0.9%, versus other, 0.03% ± 0.1%). Additionally, the short-chain fatty acid producer Pseudomonas fragi (78) showed relatively high abundance in anthocyanin-fed birds (ANTH: 0.3% ± 0.5%).

### Microbial interconnections induced by nutraceuticals.

**FIG 10:**
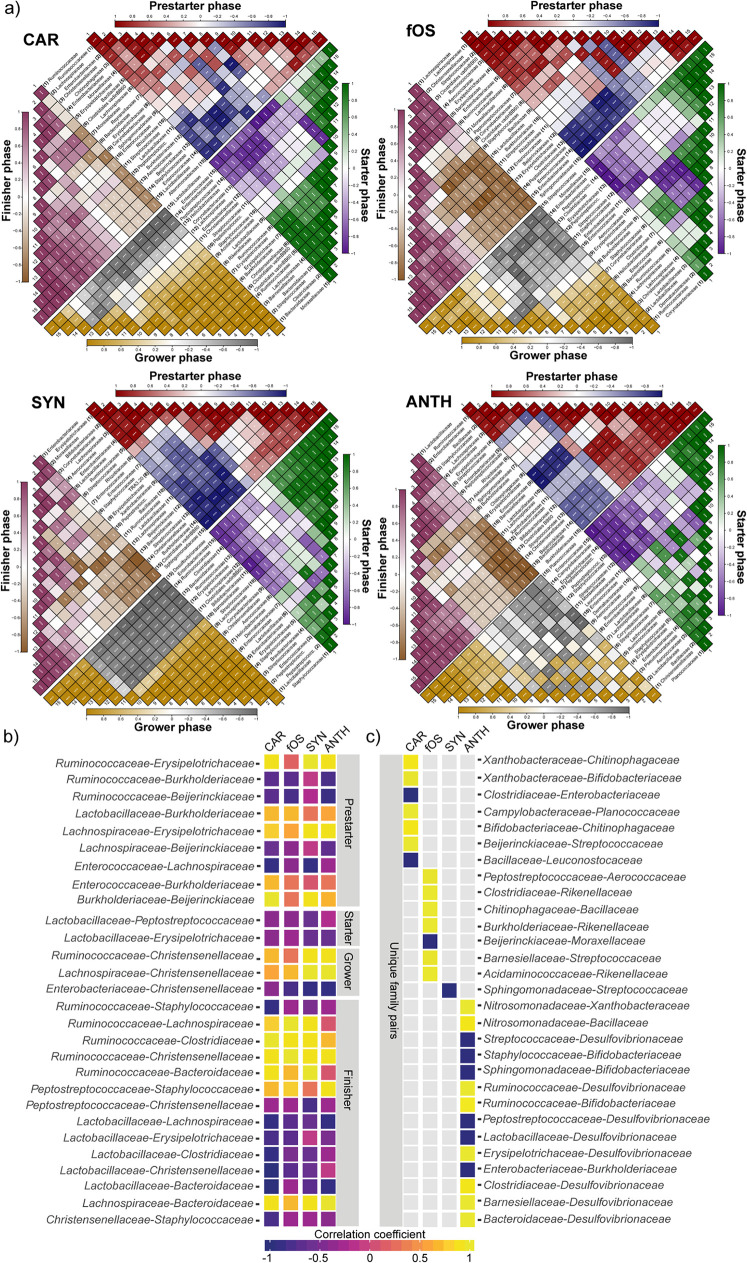
Spearman correlation plots indicating nutrient-induced interconnections between members of the broiler GIT microbiota in relation to four phases of broiler rearing. Color intensities indicate values of correlation coefficients, i.e., the strengths of associations between dedicated families. The values vary from −1 to +1, indicating the strength of positive (*r* value > 0) and negative (*r* value < 0) correlations. (a) Divergent abundancepatterns are shown by considering the taxonomy data of the 15 most abundant families in nutraceutical treatment groups throughout the four phases of the experiment. BD negative control (basal diet with no dietary supplement) and the following dietary treatments were provided as mash feed: BGLU positive control (BD including β-glucan), CAR (BD including carotenoids), fOS (BD including fermentable oligosaccharides), SYN (BD including synbiotics), and ANTH (BD including anthocyanins). (b) Matched pairs of families showing very strong correlations in relation to aging and diet. Gradient colors represent extents of positive and negative correlations. There were 13 very strong (*r* value > 0.8) positive (prestarter phase: 5; grower phase: 2; finisher phase: 6) and 15 negative (prestarter phase: 4; starter phase: 2; grower phase: 1; finisher phase: 8) associations detected throughout the four rearing phases of the experiment. (c) Unique, diet-specific matches showing very strong correlations in family taxonomy.

To identify nutraceutical-induced interconnections within the broiler intestinal microbiota, we estimated the extent to which relevant families tended to change together. Relative proportions of taxa were correlated in terms of Spearman’s method (Fig. 10). We identified divergent abundance patterns by using data for the 15 most abundant families in nutraceutical-induced treatment groups throughout the four phases of the experiment (Fig. 10a). In general, similar correlation patterns were revealed between CAR-SYN- and fOS-ANTH-treated samples. We focused on two areas. (i) First, we attempted to find correlations between families throughout the four feeding phases of the experiment. We found 13 statistically significant positive (prestarter: 5; grower: 2; finisher: 6) and 15 negative (prestarter: 4; starter: 2; grower: 1; finisher: 8) associations throughout the experiment (Fig. 10b). (ii) Second, we identified very strong correlations between families in that were exclusive to specific diets (Fig. 10c). Characteristically, anthocyanin-treated samples showed by far the highest number of unique family matches (8 positive versus 6 negative correlations). As such, *Desulfovibrionaceae* showed very strong negative correlations with *Lactobacillaceae* (*r* value: −0.97), *Streptococcaceae* (*r* value: −0.97), and *Peptostreptococcaceae* (*r* value: −0.97) in ANTH-treated samples. Concurrently, the family *Desulfovibrionaceae* correlated very strongly with *Bacteroidaceae* (*r* value: 0.97), *Barnesiellaceae* (*r* value: 0.97), *Clostridiaceae* (*r* value: 0.97), *Erysipelotrichaceae* (*r* value: 0.97), and *Ruminococcaceae* (*r* value: 0.97) in these samples. In SYN-treated samples, a very strong negative association was found between *Sphingomonadaceae* and *Streptococcaceae* (*r* value: −1). The peculiar fingerprint of the fOS-supplemented diet showed a very strong negative association between *Moraxellaceae* and *Beijerinckiaceae* (*r* value: −0.97). In animals fed fOS, very strong positive interrelations were detected between *Rikenellaceae* and *Clostridiales* (*r* value: 0.97), *Rikenellaceae* and *Burkholderiaceae* (*r* value: 0.97), and *Rikenellaceae* and *Acidaminococcaceae* (*r* value: 0.97). Furthermore, very strong connections were detected between *Streptococcaceae* and *Barnesiellaceae* (*r* value: 0.97), *Aerococcaceae* and *Peptostreptococcaceae* (*r* value: 1), and *Chitinophagaceae* and *Bacillaceae* (*r* value: 0.97). The CAR characteristic fingerprint showed very strong positive correlations between *Xanthobacteraceae* and *Chitinophagaceae* (*r* value: 0.91), *Xanthobacteraceae* and *Bifidobacteriaceae* (*r* value: 1), and *Beijerinckiaceae* and *Streptococcaceae* (*r* value: 0.97). Additionally, the family *Bifidobacteriaceae* showed a strong positive association with *Chitinophagaceae* in CAR-treated birds (*r* value: 0.91).

## DISCUSSION

An extraction technology was developed (34) that is able to recycle plant-based food industrial waste to extract its bioactive compounds (anthocyanins from sour cherry and carotenoids and fermentable oligosaccharides from red sweet pepper) and conserve their beneficial, health-promoting effects. Based on this invention, our prior aim was to develop forage enriched in nutraceuticals and to investigate the effect of these natural feed additives on the broiler GIT microbiota.

The feeding program of this trial was applied according to the norms widely used in Ross 308 chicken production (34). Based on our findings, bioactive compound-enriched diets have been shown to strengthen the positive correlations between body weight and the beneficial orders *Bacillales*, *Rhizobiales*, and *Corynebacteriales*, which are associated with increased nutrient absorption through the improvement of the intestinal epithelium integrity (79, 80). We found that, under our experimental conditions, a nutraceutical-enriched diet did not significantly improve body weight, supporting the estimations of other data (81, 82). Additionally, our data did not support that probiotics enhance animal growth, which might be explained by a number of different environmental and genetic factors (36). Nutraceuticals did not significantly increase the relative proportions of *Lactobacillaceae* and *Bifidobacteriaceae*, which were previously reported to amend the utilization of prebiotic oligosaccharides in chicken (24, 83–86). Furthermore, we theorize that the noticeable decrease in intestinal *Clostridium* and *Bacteroides* of anthocyanin-treated birds may be associated with alterations in bile biotransformation through which the microbiota impacts host fat digestion and utilization. Notably, we did not observe any decrease in the body gain rate of anthocyanin-treated birds (ANTH finisher phase, 2,590 ± 280 g, versus BD, 2,758 ± 264 g).

A combined age-related view of the healthy, baseline GIT microbiota was also achieved at the phylum, order, and genus taxonomic ranks of baseline bacteria at different stages of Ross 308 broiler production. This showed that the broiler GIT microbiota was dominated by two core phyla: *Firmicutes* (93.0% ± 6.9%) and *Proteobacteria* (6.9% ± 0.9%).

We also investigated the effects of different dietary supplements on GIT community complexity through the production of Ross 308 Gallus gallus *forma domestica*. Based on our results, remarkable increases were detected in Faith’s index due to fOS, SYN, and ANTH diet in relation to those of both controls (BD, BGLU). According to our estimations, the fOS-supplemented diet increased Faith’s index, which was consistent with the results reported by Shang et al. (35). Furthermore, in accordance with a previous study (87), we found that carotenoids did not exert significant effects on community complexity. Probiotics are increasingly applied to animals in poultry industries, too (39, 88). Additionally, based on our findings, β-glucan supplementation did not exert a remarkable influence on community diversity. Similar to previous reports, our data indicated that the composition of the broiler GIT microbiota diversifies remarkably as the GIT microbial population becomes more complex in aging broilers (39, 89). An increase in community alpha diversity makes symbiotic communities more discordant, which was also supported by Bray-Curtis, Jaccard, and weighted and unweighted UniFrac distances. Notably, the present study revealed that appreciable beneficial effects of nutraceuticals manifested mostly by the end of the broiler productive life span, as the diversity started to decrease. This may suggest that dietary supplementation has a lesser impact on a more diverse symbiotic microbiota. Higher microbial diversity is commonly related to a healthier host status, whereas a lack of sufficient diversity in microbial community structures has been associated with intestinal diseases (10, 90–94). Furthermore, imbalance of the gut microbiome composition and significant losses in GIT diversity often lead to the elimination of beneficial bacteria and accompanying increases in pathogenic bacteria (95).

Additionally, we managed to investigate how nutraceuticals can shift the abundances of potential zoonotic strains. The final 2 weeks of the broiler production period is associated with elevated mortality and production losses due to localized or systemic bacterial infections. In addition to the genetic background, the performance and meat production of domestic animals (e.g., broilers) are influenced by water and feed quality, energy and nutrient content of the diet, and their relative proportions, as well as various environmental factors (ambient temperature, humidity, air speed, ventilation technique, herd density in the barn, and, moreover, environmental stress) (96, 97).

Identifying symbiotic and dysbiotic taxa is not a straightforward task, and there are no obvious “good or bad guys” in complex microbial communities. However, it is essential to consider the problem of livestock contamination for both sanitation and economic reasons (85). In our experimental system with 1,080 animals, the mortality rate proved to be very low (0.56%); nonetheless, no significant differences in lethality patterns were observed between our experimental settings.

In this study, the *Firmicutes-*to-*Bacteroides* ratio was lowest in anthocyanin-fed animals, which was accompanied by a decrease in body weight in comparison to that of the controls. The potential pathogen genus *Bacteroides* encodes a high number of proteins involved in polysaccharide and monosaccharide metabolism, decreases colonic pH, and improves the function of epithelial cells (98). The increase in *Bacteroides* frequencies in the starter flock due to synbiotics supposedly modulated their polysaccharide metabolism since members of this genus are generally associated with the degradation of starch and glucan (76). However, these suggestions were not supported by our data. Acetate and propionate are mainly produced by *Bacteroidetes*, while *Firmicutes* are the main butyrate supplier (37, 99, 100). The highest ratios for Bacteroides gallinaceum were detected in samples receiving carotenoids and anthocyanins, while Bacteroides dorei was traceable only in CAR-, fOS-, and SYN-fed birds. Notably, in prestarter and finisher broilers, anthocyanins increased the levels of the beneficial bacteria *Lachnospiraceae* and *Ruminococcaceae*, which are usually associated with improvements in feed conversion (51). Furthermore, during the finisher phase, anthocyanins increased the levels of *Akkermansiaceae*, *Bacteroidaceae*, and *Barnesiellaceae*, which are in turn linked to more efficient intestinal absorption of compounds, as described previously (101). This might be suggestive of improvements in growth parameters; however, these were also not strengthened by our data.

The beneficial effects of nutraceuticals manifested in the increasing proportions of the butyrate producers *Lachnospiraceae* and *Ruminococcaceae* in finisher chickens. For colonocytes, butyrate is an important energy source that is largely metabolized in the epithelial mucosa (102). Mucin-degrading *Akkermansia* species are usually associated with intestinal health, due to their competitive exclusion of other, less beneficial bacteria that adhere less effectively to the mucosal surface (103, 104). Additionally, *Akkermansia* was previously shown to decrease visceral fat deposits; thus, their abundance might be associated with decreases in body weight gain (103–105). However, in this study, no significant associations were found between *Akkermansia* and broiler weight. Anthocyanins enhanced the frequencies of the important butyrate producer genus *Eubacterium* (40, 106, 107), while fermentable oligosaccharides and synbiotics increased the relative abundance of the genus *Clostridium* during the prestarter feeding period, which might be associated with beneficial effects on animal GIT health (108).

In addition to involvement in carbohydrate metabolism, some members of the genera *Helicobacter*, *Clostridium*, and *Enterococcus* are important pathogens (86) that colonize the gastrointestinal tract of chickens, causing gastroenteritis (73), necrotic enteritis (24, 25), and enterococcal spondylitis (72). Notorious members of the genus *Clostridium* also have beneficial physiological effects on various biological responses by synthesizing essential vitamins and micronutrients (thiamine, riboflavin, nicotinamide, pantothenic acid, biotin, etc.), neurotransmitters (biogenic amines), and secondary bile acids for the host (102, 109, 110). Furthermore, certain members are also known polyphenol producers, exhibiting antioxidant activity and decreasing inflammation (111). Lipoglycans of *Clostridium* and *Enterococcus* spp. are known to trigger inflammatory responses and insulin resistance (112). In the case of *Clostridium*, the highest ratios were noted in prestarter birds, treated with β-glucan, fermentable oligosaccharides, and synbiotics, whereas the highest abundances of *Enterococcus* were registered in prestarter and starter birds where nutraceuticals, especially fermentable oligosaccharides and anthocyanins, boosted their frequencies in comparison to controls. Previous studies reported decreased *Campylobacter* and *Clostridium* colonization measured in broilers fed fructans (113). According to our data, the proportion of the family *Campylobacteraceae* was significantly decreased in finisher animals receiving immunostimulants relative to those receiving the basal diet. In carotenoids-fed birds, *Eggerthella* increased remarkably, whereas immunostimulants (BGLU and nutraceuticals) were able to decrease the abundances of the genus *Helicobacter*. Interestingly, in chickens fed anthocyanins, a noticeable increase was registered for the bacterial diarrheal gastroenteritis-causing C. jejuni without affecting chicken welfare. Of note, C. jejuni can also be involved in the maintenance of intestinal epithelial integrity and the modulation of anti-inflammatory and antitumor effects (35, 57, 114). Although the specific mechanisms have not been fully elucidated, phytonutrients rich in antioxidants can reduce pathogenic stress (115). The Gram-negative, rod-shaped, opportunistic pathogen Alcaligenes faecalis, which can trigger infections by colonizing the respiratory tract (116), was not traceable in broilers receiving either β-glucan or nutraceuticals.

The most widely used probiotics are members of the relevant acetate-producing genus *Lactobacillus* (22, 117), which has also been reported to positively affect the gut health of poultry by reducing inflammation and controlling enteric bacterial infections through regulating mucin composition (16, 17, 75, 99). In this trial, carotenoids were shown to positively modulate the abundances of the genus *Lactobacillus* in grower and finisher animals, which might also affect certain enzymatic activities of the oligosaccharide transport system of lactobacilli (118). These data are consistent with the results of other studies reporting *Lactobacillus* as a major beneficial bacterium showing increases in broilers fed fructans (37, 38). In control samples, elevated levels were measured for Lactobacillus salivarius in relation to that in treatment groups, which can be associated with enhanced induction of anti-inflammatory responses of chicken (99). Furthermore, the age-related oscillating patterns of the genus *Lactobacillus* might also be congruent with deconjugated bile acid concentrations in broiler chickens (51, 119). Both human and animal studies found an association between the accumulation of lactic acids and disease states, such as colitis and gut resection (120, 121). In our study, taxonomic heat trees indicated that anthocyanins remarkably decreased the relative abundance of the family *Lactobacillaceae*.

The most pronounced negative correlations between butyrate-producing genera such as *Butyricicoccus* and *Ruminococcus* and lactic acid-producing *Lactobacillus* have been revealed in anthocyanin-treated animals. According to our assumptions, this might be associated with improvements in epithelial intestinal barrier functions that are caused by decreasing lactic acid buildup and increasing osmotic load (122). Interestingly, a strong negative correlation was revealed between the lactate- and acetate-producing *Bifidobacteriaceae* and lactic acid-producing *Staphylococcaceae* (*r* value: −0.97) in animals fed anthocyanins. In finisher animals, very strong negative correlations were detected in birds fed nutraceuticals between *Lactobacillaceae* and *Bacteroidaceae*, whose members are known to improve metabolic efficiency and reduce colonization by undesirable microbes (36, 117, 120).

### Conclusions.

We report the following main results based on our data. (i) Time exerted a great influence on the chicken microbial community structure. There was a tendential increase in broiler GIT community diversity as chickens aged. Subsequent deviation from diversity can be alleviated by treating birds with fermentable oligosaccharides, synbiotics, and anthocyanins. (ii) Great emphasis was also placed on how taxonomy data correlate with enhanced bird body weight. Nutraceuticals resulted in strong positive correlations between body weight gain and the orders *Bacillales*, *Corynebacteriales*, *Enterobacteriales*, *Micrococcales*, and *Pseudomonadales*. (iii) The 50% core taxonomy data revealed the relations between the symbiotic broiler Ross 308 microbiota and age and diet. Fermentable oligosaccharides, synbiotics, and anthocyanins were shown to exert the greatest community shifts, especially during the prestarter and starter phases. (iv) In general, *Enterobacteriaceae* (prestarter, starter), *Akkermansiaceae* (finisher), *Brevibacteriaceae* (starter, finisher), *Staphylococcaceae* (prestarter), *Bacteroidaceae* (starter, grower), *Bifidobacteriaceae* (starter, grower), *Campylobacteraceae* (grower, finisher), *Helicobacteraceae* (finisher), *Planococcaceae* (grower, finisher), and *Pseudomonadaceae* (grower, finisher) were identified as key taxa representing significant shifts (mean log_2_ fold change |≥2|) in community taxon compositions due to nutraceuticals. (v) There were alterations in relative frequencies of commensal beneficial, short-chain fatty acid-producer bacteria and conditioned pathogens. The *Firmicutes*-to-*Bacteroides* ratio (F/B) proved to be the highest in β-glucan-treated animals and the lowest in anthocyanin-treated animals. Coincidentally, anthocyanins were shown to increase *Faecalibacterium*, *Blautia*, and *Ruminococcus* in finisher birds remarkably relative to BD. Generally, fermentable oligosaccharides, synbiotics, and anthocyanins exerted a positive impact on *Faecalibacterium*, and the difference was more pronounced by the end of broiler rearing. Impressive alterations in *Lactobacillus* were mostly age related. Carotenoids were shown to increase *Bifidobacteriaceae* and *Barnesiellaceae* but reduce *Enterococcaceae* and *Clostridiaceae* in grower phase. (vi) Spearman’s correlations identified mutual interconnections, i.e., very strong age- and diet-related associations of the symbiotic broiler gastrointestinal microbiota. Very strong positive correlations were revealed between body weight and the families *Campylobacteraceae-Planococcaceae* (CAR), *Streptococcaceae-Beijerinckiaceae* (CAR), *Peptostreptococcaceae-Aerococcaceae* (fOS), *Burkholderiaceae-Rikenellaceae* (fOS), *Bacillaceae-Nitrosomonadaceae* (SYN), *Ruminococcaceae-Bifidobacteriaceae* (ANTH), and *Clostridiaceae-Desulfovibrionaceae* (ANTH) for individual nutraceuticals.

This is a unique and comprehensive trial that highlights the health benefits of bioactive compounds of recycled food waste products as potential dietary adjuncts for antibiotic-free broiler meat-production systems. Based on our observations, a nutraceutical-enriched diet did not degrade chicken development and delivered promising results in stimulating GIT health.

Additionally, this study also improves our knowledge about the effects of carotenoids, fermentable oligosaccharides, anthocyanins, and synbiotics on the composition of the broiler gastrointestinal tract microbiota.

## MATERIALS AND METHODS

### Birds and housing.

A total of 1,080, 1-day-old Ross 308 mixed-sex broilers from a commercial hatchery in Hungary were used. The experiment was carried out on the experimental farm of the University of Debrecen. All broilers were housed in the same shed. Chickens were kept in floor pens covered with wood shavings in a thermostatically controlled house at a stocking density of 650 cm^2^/bird and reared under standard management conditions. Sampling procedures were carried out in accordance with the local (University of Debrecen) ethics committee’s approved guidelines (DEMAB/12-7/2015).

### Experimental design and dietary treatments.

One-day-old Ross 308 hybrid chicks were randomly placed into 6 experimental groups (3 replicates/treatment, 60 birds/pen). The experiment was started at day 1 of age and lasted until 42 days. Each group was fed one of the following 6 diets: basal diet (BD), without any added supplements; basal diet including 0.5% β-glucan (BGLU); basal diet including 0.5% carotenoids (CAR); basal diet including 0.5% fermentable oligosaccharides (fOS); basal diet including 0.5% synbiotics (SYN); basal diet including 0.5% anthocyanins (ANTH). BD (negative) and BGLU (positive) were the control groups, and CAR, fOS, SYN, and ANTH were the treatment groups. Broilers were fed a commercial maize-soybean-based basal diet (BD) free of antibiotics according to four feeding periods: prestarter (1 to 9 days), starter (10 to 21 days), grower (22 to 31 days), and finisher (32 to 42 days). All diets were fed in mash form. The compounds and nutritional composition of BD are given in Table 1. The composition of nutrients in each basal diet was planned to satisfy nutritional requirements of broiler chickens according to the National Research Council (NRC) (123). Feed and water were available *ad libitum* during the entire experiment. Broilers were weighed at 1, 10, 21, 32, and 42 days of age. As growth performance parameters, average body weight (BW) was calculated. Mortality was monitored; it was very low (0.56%), and there was no association between mortality and feed treatments. No veterinary treatment was required for the entire duration of the experiment.

**TABLE 1:** Ingredients and chemical composition of the basal diet

Ingredients^*a*^	Diets			
	Prestarter (day 1–9)	Starter (day 10–21)	Grower (day 22–31)	Finisher (day 32–42)
Corn, %	33	34	33	32
Wheat, %	27	29	31	32
Soybean meal, solvent extracted (46.0% CP), %	29	24	20	16
Soybean meal, extruded (46.0% CP), %	4	6	4	4
Sunflower meal, extracted, %	-	1	3	4
Feed yeast, %	1	-	-	-
Distillers’ dried grains with solubles, %	-	1	3	5
Plant fats, %	2	1	3	4
Premix, %	4	4	3	3
Total, %	100	100	100	100
**Energy and nutrient contents of the diets**				
Dry matter, %	89.06	89.03	89.15	89.15
AME_n_ poultry, MJ/kg	12.23	12.47	12.81	13.01
Crude protein, %	21.58	20.28	19.05	18.28
Crude fat, %	4.61	4.83	6.22	6.83
Crude fiber, %	3.37	3.51	3.7	3.88
Lysine, %	1.37	1.27	1.17	1.09
Methionine, %	0.57	0.54	0.53	0.49
Methionine + cysteine, %	0.94	0.9	0.87	0.83
Calcium, %	0.85	0.73	0.71	0.67
Phosphorus, %	0.63	0.55	0.52	0.49
Phosphorus utilization, %	0.45	0.42	0.40	0.35
Sodium, %	0.17	0.16	0.16	0.16
Sodium chloride, %	0.282	0.252	0.242	0.244
Vitamin A, mg/kg	12,500	12,500	12,500	8,750
Vitamin D_3_, mg/kg	3,000	3,000	3,000	2,100
Vitamin E, mg/kg	50.001	50.001	50.001	35
Lasalocid sodium, mg/kg	82.500	82.500	82.500	

a CP, crude protein; AME_n_, apparent metabolizable energy, n = corrected for zero nitrogen balance.

### Determination of natural feed additives.

Carotenoid (CAR) supplementation was determined as described by Remenyik et al. (124) and Csernus et al. (34) (see Fig. S1 in the supplemental material). Carotenoids were extracted from Hungarian red sweet pepper powder (in 1 to 5 g) using dichloroethane-acetone-methanol as the solvent mixture in a 2:2:1 ratio. The mixture was agitated in an ultrasonic water bath for 30 min and purified through Munktell-292 filter paper (VWR International, Debrecen, Hungary). For further purification, a 0.22-μm polytetrafluoroethylene (PTFE) syringe filter (TPP Techno Plastic Products AG, Switzerland) was applied. Afterward, the filtered sample was vaporized at 40°C at 20 kPa and then dissolved in a high-performance liquid chromatographic (HPLC) reagent (isopropanol-acetonitrile-methanol at 55:35:10) (Merck, Darmstadt, Germany). HPLC separation was conducted on a Phenomenex Kinetex column (2.6 μm, XB-C_18_, 100 Å, 100 × 4.6 mm) (Phenomenex, Torrance, CA, USA) with the following two solvents: A, 11% methanol, and B, isopropanol-acetonitrile-methanol (55:35:10, vol/vol/vol %). Step elution was performed with the following settings: 0 to 3 min 100% solvent A, 15 to 20 min 20% solvent A, 25 to 45 min 100% solvent B, and 48 to 50 min 100% solvent A. For detection, a diode array detector (DAD) and a 0.6-ml/min flow rate were applied. The sample was injected in a 10-μl volume, and the DAD detection was applied at 460 and 350 nm. The HPLC profile and carotenoid compounds with the greatest areas are provided in the supplemental material (Fig. S1).

10.1128/mSystems.01124-20.1 FIG S1 Determination of carotenoids: UHPLC profile of carotenoid supplement (CAR) and table with carotenoid compounds with retention times and relative percentage of areas. Download FIG S1, PDF file, 0.2 MB. Copyright © 2021 Tolnai et al. 2021 Tolnai et al. https://creativecommons.org/licenses/by/4.0/ This content is distributed under the terms of the Creative Commons Attribution 4.0 International license.

Fermentable oligosaccharide (fOS) supplementation was performed as described in the work of Csernus et al. (34) (Fig. S2). Hungarian red sweet pepper was also applied to extract fermentable oligosaccharides (fOS) with high arabinogalactose content. To assess the composition of oligosaccharides, an HP 5890 gas chromatograph (GC) was applied with an SP-2380 capillary column (30 m by 0.25 mm, 0.2 μm). Samples were lyophilized and extracted with trifluoracetic acid-acetic acid-water (5:75:20) as the solvent. Oligosaccharides were turned into alditol-acetate. After the reduction step, sugars were shifted to sugar alcohols (alditols), which removed interfering isomers and anomers. Reduction was performed with NaBH_4_ at alkaline pH. Acetylation was also performed with acetic anhydride in pyridine. The feed gas was nitrogen at a 1.2-ml/min flow rate. The injector temperature was set to 300°C, and split ratio was 1:20. A flame ionization detector (FID) was used for identification of oligosaccharides. The GC profile and the identified monomer units of oligosaccharides are provided in the supplemental material (Fig. S2).

10.1128/mSystems.01124-20.2 FIG S2 Determination of fermentable oligosaccharides: Gas chromatograph profile of monomer units of fermentable oligosaccharide supplement (fOS) and table identifying fermentable oligosaccharide monomers with retention times and relative percentage of areas. Download FIG S2, PDF file, 0.08 MB. Copyright © 2021 Tolnai et al. 2021 Tolnai et al. https://creativecommons.org/licenses/by/4.0/ This content is distributed under the terms of the Creative Commons Attribution 4.0 International license.

The synbiotic (SYN) supplement contained probiotics (Bifidobacterium bifidum, Bifidobacterium infantis, Bifidobacterium lactis, Bifidobacterium longum, Lactobacillus acidophilus, Lactobacillus buchneri, Lactobacillus casei, Lactobacillus paracasei, Lactobacillus plantarum, *L. salivarius*, and Lactobacillus lactis), prebiotics (fructo-, xylo-, and mannooligosaccharides and arabinogalactan) (Fig. S3), vitamins (B group vitamins and vitamins C, D_2_, D_3_, E, and K_2_), unsaturated fatty acids (ω-3, ω-6, and ω-9), mineral and trace elements (sodium, potassium, calcium, iodine, and phosphorus), and lactose. The GC profile and the identified monomer units of oligosaccharides are provided in the supplemental material (Fig. S3).

10.1128/mSystems.01124-20.3 FIG S3 Determination of synbiotics: gas chromatograph profile of monomer units of synbiotic supplement (SYN) and table identifying oligosaccharide monomers with retention times and relative percentage of areas. Download FIG S3, PDF file, 0.08 MB. Copyright © 2021 Tolnai et al. 2021 Tolnai et al. https://creativecommons.org/licenses/by/4.0/ This content is distributed under the terms of the Creative Commons Attribution 4.0 International license.

Anthocyanin (ANTH) supplementation was determined as described by Nemes et al. (125) (Fig. S4). Anthocyanins were extracted from Hungarian sour cherry. Cherries were deseeded and homogenized, and then methanol-water-acetic acid solution in a 25:24:1 ratio was utilized to extract anthocyanins. The sample was mixed with a magnetic stirrer (MSH 300, BioSan, Riga, Latvia) for 1 h. Filtering and centrifugation were performed at 10,000 rpm for 5 min, and then a simple fractionation was carried out in preconditioned tubes (Superclean ENVI-18 SPE tubes). For preconditioning, 5 ml of methanol (MeOH), 5 ml of H_2_O, and 1 ml of fruit sample were used. The elution was conducted with methanol containing 20% H_2_O and vaporized at 40°C. The sample was dried in vacuum to powder. A VWR-Hitachi ChromasterUltraRs ultra-HPLC (UHPLC) instrument (Hitachi, Tokyo, Japan) was used for anthocyanin profile determination with a Phenomenex Kinetex column (2.6 μm, XB-C_18_, 100 Å, 100 × 4.6 mm) (Phenomenex, Torrance, CA, USA). Two solvents were applied for a step elution, A (MeOH) and B (3% formic acid), with the following parameters: 0 min, 15% solvent A; 0 to 25 min, 30% solvent A; 25 to 30 min, 40% solvent A; and 30 to 40 min, 50% solvent A. UV-visible (UV-VIS) detection was applied at 534 nm, the flow rate was kept at 0.7 ml/min at 25°C, and the injection volume was 10 μl. The UHPLC profile and the main anthocyanin compounds are included in the supplemental material (Fig. S4).

10.1128/mSystems.01124-20.4 FIG S4 Determination of anthocyanins: UHPLC profile of anthocyanin supplement (ANTH) and table identifying anthocyanin compounds with retention times and relative percentage of areas. Download FIG S4, PDF file, 0.1 MB. Copyright © 2021 Tolnai et al. 2021 Tolnai et al. https://creativecommons.org/licenses/by/4.0/ This content is distributed under the terms of the Creative Commons Attribution 4.0 International license.

### Sample collection.

Fecal samples were collected at 7, 19, 31, and 40 days of age (prestarter, starter, grower, and finisher sampling periods, respectively). In every experimental group (BD, BGLU, fOS, CAR, SYN, and ANTH), 4 fecal samples (1 pullet and 1 cockerel, 2 fecal pools) were collected over the whole experimental period. Fecal samples were collected freshly into specific, sterile, DNase-free stool transportation bowls and immediately placed on ice for a maximum of 3 h. Unprocessed samples were kept at −80°C until further use.

### Sample preparation and mechanical cell lysis.

Bacterial cell suspensions (BS) were prepared from 7 g of each broiler stool sample. Then, 7 ml of sterile PBS buffer (Thermo Fisher Scientific, MD, USA) was added to each of the samples, and they were homogenized for 4 min (by vortexing at 350 rpm) (126). The samples were centrifuged for 5 min at 500 × *g*. Supernatants were collected, and the washing step was repeated 2 times. Supernatants were centrifuged for 20 min at 13,000 × *g*. Finally, the supernatants were discarded, and the bacterial pellets were dissolved in 3 ml of sterile PBS buffer. One-milliliter aliquots of BS were added to PowerBead tubes (Qiagen, Hilden, Germany) for mechanical cell lysis. Bacterial cell disruption was performed with a MagNA Lyser instrument (Roche Applied Sciences, Penzberg, Germany) set to 5,000 rpm for 30 s.

### DNA extraction.

Total bacterial genomic DNA was extracted with the conventional isolation method. A total of 800 μl of sample lysate was mixed with 800 μl of phenol-chloroform-isoamyl alcohol (25:24:1) (Thermo Fisher Scientific, MD, USA) and vortexed thoroughly for approximately 20 s. After homogenization, the samples were incubated at room temperature for 3 min and centrifuged for 10 min at 16,000 × *g*. After phase separation, the upper aqueous layer was carefully collected into a new sterile DNase- and RNase-free Eppendorf tube. For DNA precipitation, a mixture of 1 μl of glycogen (20 μg), 7.5 M NH_4_OAc (ammonium acetate in 0.5× volume of the sample), and 100% EtOH (ethanol in 2.5× the volume of the sample) was added to the supernatant. The samples were incubated at −20°C overnight and then centrifuged for 30 min at 16,000 × *g* at 4°C to pellet the DNA. The supernatant was carefully discarded without disturbing the pellet, and 70% EtOH was added to the sample and shaken by hand for 20 s. Then, the samples were centrifuged at 4°C for 5 min at 16,000 × *g*, and the supernatant was carefully removed. This washing step was repeated 2 times. The DNA pellet was dried at room temperature and then resuspended in 40 μl of nuclease-free water. DNA concentrations were determined using a Qubit fluorometric quantitation double-stranded DNA (dsDNA) assay kit (Thermo Fisher Scientific, Waltham, MA, USA) on a Clariostar microplate reader (BMG Labtech, Ortenberg, Germany). DNA quantity and quality were ascertained using a NanoDrop 2000 spectrophotometer (Thermo Fisher Scientific). DNA integrity (shearing/fragmentation) was measured on a 4200 TapeStation system (G2991AA; Agilent Technologies, Santa Clara, CA, USA). The eluted DNA samples were stored at −20°C.

### Negative and positive DNA purification controls.

To minimize laboratory contamination, sterile surgical gloves and face masks were used and all DNA extraction steps were performed with sterile or sterilized equipment under a class II laminar airflow cabinet. Negative isolation control (NIC) experiments were simultaneously conducted by substituting samples with PCR-grade water. Eluted NIC samples were used for V3-V4 PCR, and indexing was performed under DNA-free UV-sterilized AirClean PCR workstations/cabinets. At each PCR cleanup step of the library preparation, NIC amplicons were also validated on a 4200 Tape Station system (G2991AA; Agilent Technologies, Santa Clara, CA, USA) using Agilent D1000 ScreenTape (5067-5365) and Agilent genomic DNA (gDNA) reagents. Host background nucleic acid contamination was also monitored with real-time PCR using the glyceraldehyde-3-phosphate dehydrogenase (GAPDH) (Sigma-Aldrich, Missouri, USA) forward primer 5′-GTCTCCTCTGACTTCAACAGCG-3′ and reverse primer 5′-ACCACCCTGTTGCTGTAGCCAA-3′ on eluted gDNAs (126).

### Library construction and sequencing.

Standard library preparation was performed according to the Illumina (San Diego, CA, USA) 16S metagenomic sequencing library preparation protocol (15044223 Rev. B). The V3 and V4 hypervariable regions of the bacterial 16S rRNA gene were sequenced with an Illumina MiSeq benchtop sequencer generating amplicons of ∼460 bp by using universal primers (341F-5′ CCTACGGGNGGCWGCAG 3′ and 785R-5′ GACTACHVGGGTATCTAATCC 3′ flanked by Illumina overhang adaptor sequences [forward overhang: 5′-TCGTCGGCAGCGTCAGATGTGTATAAGAGACAG-3′; reverse overhang: 5′-GTCTCGTGGGCTCGGAGATGTGTATAAGAGACAG-3′] [Sigma-Aldrich, Missouri, USA]). After completion of the PCR with 2× KAPA HiFi HotStart ReadyMix, dual indexing of the samples with adaptor sequences (i7-N7xx-12 and i5-S5xx-8) was performed using the Illumina (San Diego, CA, USA) Nextera XT index kit (FC-131-1001/2). PCR cleanups and amplicon size selections were carried out with KAPA Pure Beads (KAPA Biosystems) based on the technical data sheet (KR1245-v3.16) of the manufacturer, resulting in final libraries with entries of ∼580 to 630 bp. Every time, verifications were performed with PCR Agilent D1000 Screen Tape (5067-5582) and D1000 reagents (5067-5583). The 16S amplicon libraries for each sample were quantified with qPCR, normalized with respect to amplicon sizes, and pooled into a single library in equimolar quantities. Finally, 5 μl of a pooled 4 nM DNA library was used for sequencing on the Illumina MiSeq platform. The library pool was denatured with 0.2 M NaOH and diluted to 8 pM. Sequencing was carried out with a MiSeq reagent kit v3-618 cycle (MS-102-3003) following the manufacturer’s protocols (Illumina, Inc., San Diego, CA, USA). Paired-end sequencing (2 × 301 nucleotides [nt]) was performed on an Illumina MiSeq platform with a 5% PhiX spike-in quality control (PhiX control kit v3-FC-110-3001).

### Sequence processing and analysis.

Illumina BaseSpace software was used to demultiplex the paired-end reads and construct FASTQ files. The sequencing data were analyzed using Quantitative Insight Into Microbial Ecology (QIIME 2, v 2019.7) (127). Adaptor sequences (CTGTCTCTTATACACATCT) were found and trimmed from the 3′ end of the reads with Cutadapt software integrated in the QIIME 2 pipeline. DADA2 software was used for quality trimming and filtering and for chimera removal. Sequences were clustered into amplicon sequencing variants (ASVs), with 97% similarity in sequences (128). The trimming parameters were set as follows: for the forward reads, 1 base was cropped from the start and the length was set to 300 bases; for the reverse reads, 9 bases were cropped from the start of the reads and the length was set to 223 bases.

### Bioinformatic analyses.

Multiple sequence alignment was performed with the MAFFT software (129), and reads were taxonomically classified using the naive Bayesian classifier trained on the SILVA (ver132) (130) reference database by selecting mapping points according to the forward-reverse primer set that was used for amplifying the 16S rRNA V3-V4 regions of the bacterial community (341F, 806R). Phylogenetic trees were constructed with the FastTree plugin (131). The QIIME2 pipeline was applied to perform alpha and beta diversity tests. For sample normalization, an 11,500 read depth was set. In the case of alpha diversity, Shannon’s index (132), Faith’s phylogenetic diversity index (133), Simpson evenness (134), and the Chao-1 index (135) were calculated in the QIIME2 pipeline. For beta diversity analysis, weighted/unweighted UniFrac distances (136) and Bray-Curtis dissimilarities (137) were measured. Alpha diversity differences were compared using the Kruskal-Wallis test. Beta diversity group significance was calculated with permutational multivariate analysis of variance (PERMANOVA) pseudo-F statistical test. These statistical tests were used to compare diversity between treatments; significance was *P* < 0.05. QIIME2 artifact files were exported from the pipeline and converted to TSV files that were used with different visualization packages. Heatmaps were generated in Python (ver3.6.5) with the Seaborn package (0.10.0); area and donut plots were constructed with pandas (0.25.3) and matplotlib (3.1.3) packages. Boxplots, violin plots, and line plots were constructed using GraphPad Prism statistical software. R (v 3.6.2) was used to visualize bubble plots and polar plots. A differential heat tree was created with the Metacoder R package (138). In the case of differential heat trees, differences were determined using a Wilcoxon rank sum test. LEfSe analysis was performed with bioBakery tools developed by the Huttenhower lab (139). Spearman correlation matrices were calculated and visualized with R statistical software using the corrplot package (https://github.com/taiyun/corrplot).

### Data availability.

All sequence data used in the analyses were deposited in the Sequence Read Archive (SRA) (http://www.ncbi.nlm.nih.gov/sra) under PRJNA633979. Sample IDs, metadata, and corresponding accession numbers are summarized in Fig. S1.
