# Supplementary material for: Nutraceuticals Induced Changes in the Broiler Gastrointestinal Tract Microbiota
Source: mSystems. 2021 Mar 2;6(2):e01124-20. doi: 10.1128/mSystems.01124-20 (PMC8546996; doi:10.1128/mSystems.01124-20)
Supplement: TABLE S1 [file msystems.01124-20-st001.pdf]

Table S1

Body weight BW (g/bird)							
	BD	BGLU	CAR	fOS	SYN	ANTH	RMSE
Day 1	38.9	37.9	38.6	38.6	39.0	38.5	0.5
Day 10	232	226	221	222	225	227	14
Day 21	759	795	769	715	742	726	38
Day 32	1713	1767.7	1709.9	1735.3	1676.6	1705.3	66
Day 42	2758	2727	2748	2618	2717	2590	98

RMSE: Root-mean-square error.
