# Supplementary material for: Nutraceuticals Induced Changes in the Broiler Gastrointestinal Tract Microbiota
Source: mSystems. 2021 Mar 2;6(2):e01124-20. doi: 10.1128/mSystems.01124-20 (PMC8546996; doi:10.1128/mSystems.01124-20)
Supplement: FIG S1 [file msystems.01124-20-sf001.pdf]

Figure S1

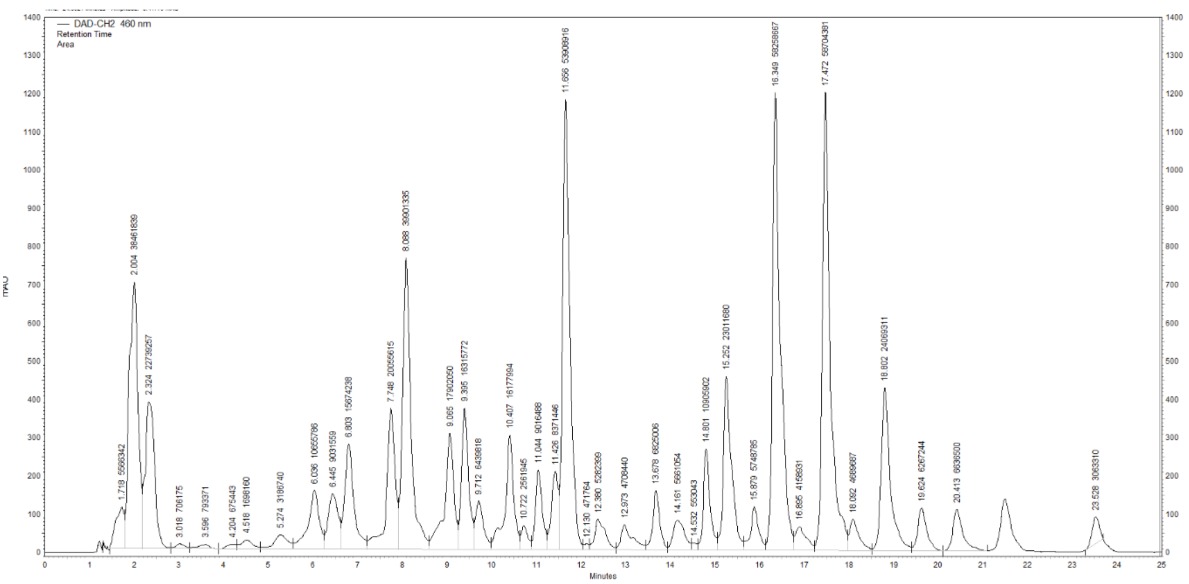

Name of carotenoids compound	Retention time (min)	Relative percentage of areas (%)
β-carotene	11.656	9.67
cis-capsanthin	16.349	10.44
capsanthin	17.472	10.54
zeaxanthin	18.802	4.39

(Y axis: Intensity of absorbance (mAU); X axis: Retention Time (min)). Table identifies carotenoids compounds with relative percentage of areas and retention times.
