# Supplementary material for: Nutraceuticals Induced Changes in the Broiler Gastrointestinal Tract Microbiota
Source: mSystems. 2021 Mar 2;6(2):e01124-20. doi: 10.1128/mSystems.01124-20 (PMC8546996; doi:10.1128/mSystems.01124-20)
Supplement: FIG S2 [file msystems.01124-20-sf002.pdf]

**Figurure S2**

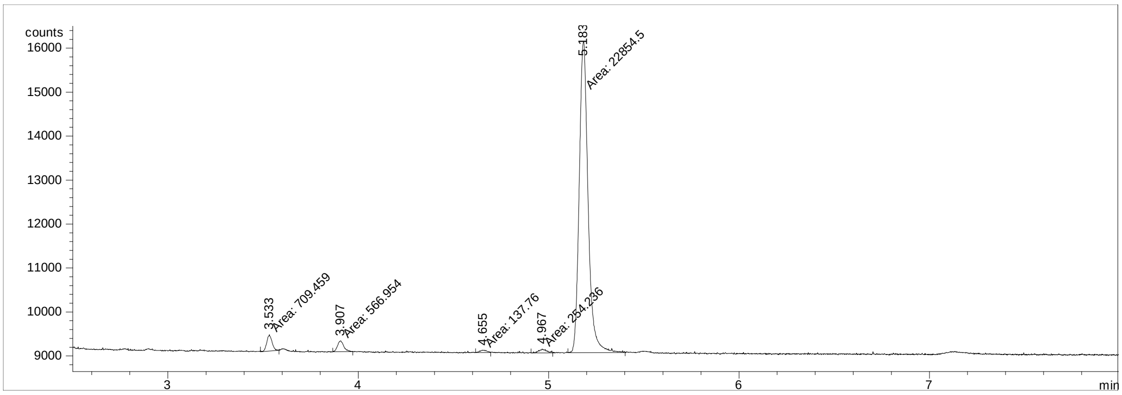


| Name of oligosaccharide monomers | Retention time (min) | Relative percentage of areas (%) |
|---|---|---|
| Arabinose | 3.533 | 2.893 |
| Xylose | 3.907 | 2.312 |
| Mannose | 4.655 | 0.562 |
| Galactose | 4.967 | 1.037 |
| Mannose | 5.183 | 93.197 |

(Y axis: counts; X axis: Retention Time (min)). Table identifies  fermentable oligosaccharide monomers with relative percentage of areas and retention times.
