# Supplementary material for: Nutraceuticals Induced Changes in the Broiler Gastrointestinal Tract Microbiota
Source: mSystems. 2021 Mar 2;6(2):e01124-20. doi: 10.1128/mSystems.01124-20 (PMC8546996; doi:10.1128/mSystems.01124-20)
Supplement: FIG S3 [file msystems.01124-20-sf003.pdf]

Figure S3

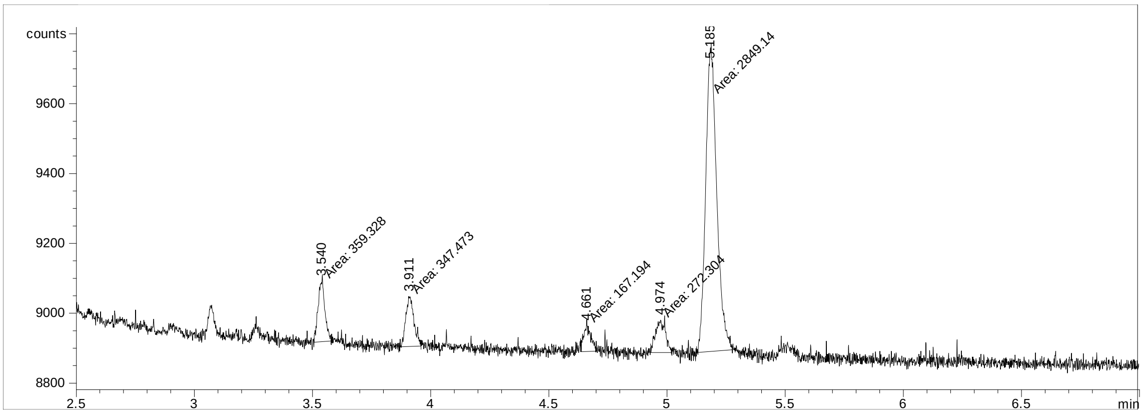

Name of oligosaccharide monomers	Retention time (min)	Relative percentage of areas (%)
Arabinose	3.54	8.993
Xylose	3.911	8.697
Mannose	4.661	4.185
Galactose	4.974	6.815
Mannose	5.185	71.31

(Y axis: counts; X axis: Retention Time (min)). Table identifies oligosaccharide monomers with relative percentage of areas and retention times.
