# Supplementary material for: Nutraceuticals Induced Changes in the Broiler Gastrointestinal Tract Microbiota
Source: mSystems. 2021 Mar 2;6(2):e01124-20. doi: 10.1128/mSystems.01124-20 (PMC8546996; doi:10.1128/mSystems.01124-20)
Supplement: FIG S4 [file msystems.01124-20-sf004.pdf]

Figure S4

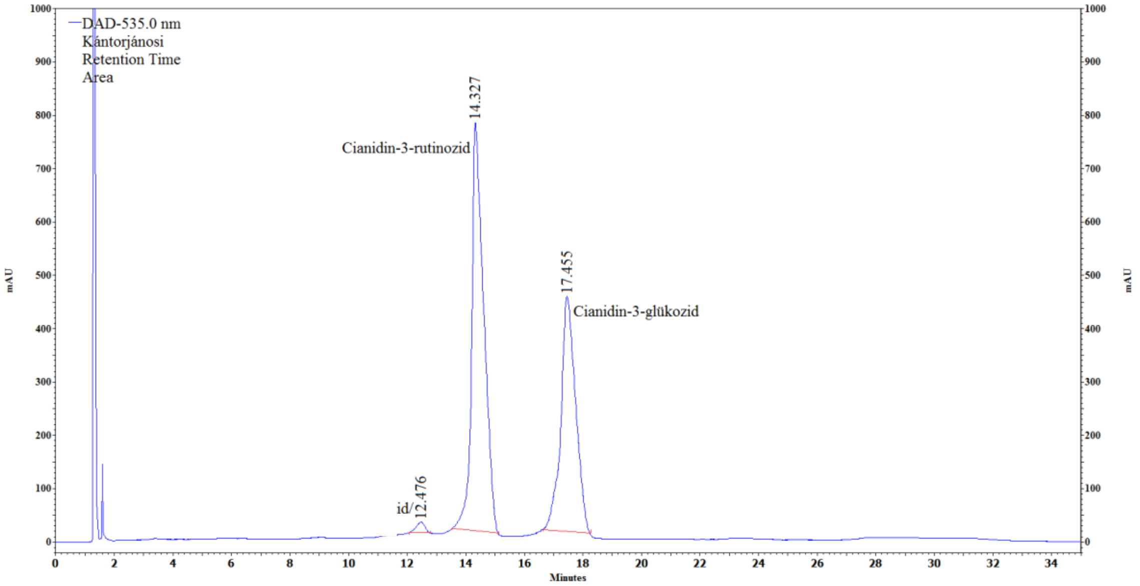

Name of anthocyanin compounds	Retention time (min)	Relative percentage of areas (%)
cyanidin-3-O-glucosyl-rutinoside	12.47	10.19
cyanidin-3-O-rutinoside	14.32	54.61
cyanidin-3-O-monoglucoside	17.45	35.19

(Y axis: Intensity of absorbance (mAU); X axis: Retention Time (min)). Table identifies anthocyanin compounds with retention areas and retention times.
